# Defect Engineering in Metal–Organic Framework Nanocrystals: Implications for Mechanical Properties and Performance

**DOI:** 10.1021/acsanm.2c00493

**Published:** 2022-03-08

**Authors:** Annika F. Möslein, Lorenzo Donà, Bartolomeo Civalleri, Jin-Chong Tan

**Affiliations:** † Multifunctional Materials and Composites Laboratory, Department of Engineering Science, University of Oxford, Parks Road, Oxford OX1 3PJ, U.K.; ‡ Dipartimento di Chimica, Università di Torino, Via P. Giuria 5, Torino 10125, Italy

**Keywords:** metal−organic frameworks, crystal growth, defects, near-field IR spectroscopy, density functional theory, mechanical properties

## Abstract

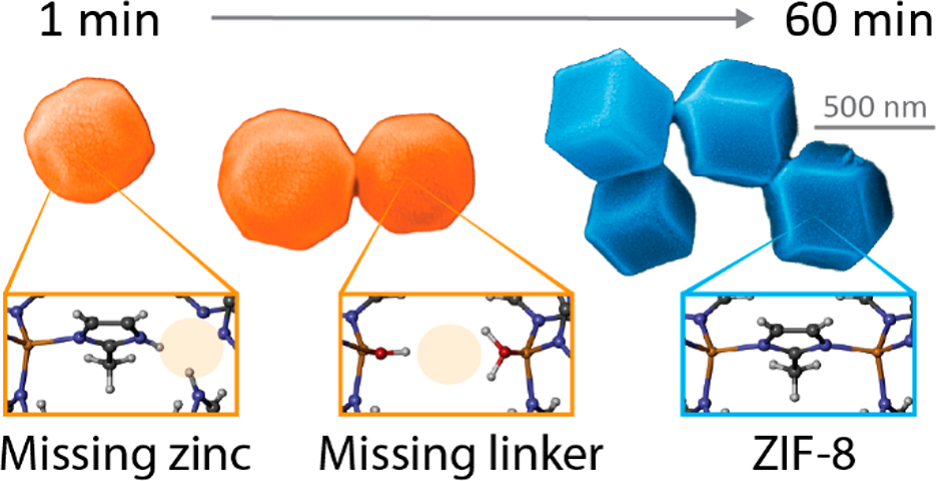

The growth process of metal–organic framework (MOF) nanocrystals defines their properties and functions. However, defects may be prevalent during the crystallization of even seemingly perfect MOFs, such as zeolitic imidazolate framework-8 (ZIF-8), and yet direct probing of such structural defects has been challenging because of the lack of nanoscale techniques to locally examine individual nanocrystals. Here, we directly study local defects, such as missing linkers or metal vacancies, in ZIF-8 nano- and microcrystals with near-field IR nanospectroscopy combined with density functional theory calculations. We track the chemical changes during crystallization and show that structural defects like zinc cations that are bound to molecules of the reactant gradually disappear with ripening of the crystals, while dangling and missing linker defects prevail. The resulting defect-terminating groups or open-metal sites produce mechanical anisotropy and reduce the Young’s modulus, as measured via tip force microscopy with nanoscale resolution and supported by theoretical modeling. However, these structural defects also open the door for defect engineering to tune the performance of ZIF-8 by offering additional adsorption sites for targeted catalytic reactions, chemical sensing, or gas capture.

## Introduction

At the nanoscale, metal–organic framework (MOF) crystals feature miscellaneous shapes and sizes. Their diversity gives rise to their vast physical and chemical properties, paving the way for applications in sensing technologies, drug delivery, gas capture, or catalysis, among others.^1−5^ This multifunctionality emerges not simply because MOFs are *per se* hybrid materials, built from metal clusters and organic linkers with a boundless number of possible combinations, but mostly because of their exceptional porosity and chemical tunability, which affords the adsorption, encapsulation, or release of versatile guest molecules.^6,7^ The power of engineering MOF nanocrystals for application is, therefore, ascribed solely to meticulous control of the framework properties and its interactions, which essentially originates in the material synthesis.^7^ For instance, the zeolitic imidazole framework ZIF-8, one of the most well-studied frameworks because of its stability and ease of synthesis, is obtained by combining zinc nitrate hexahydrate [Zn(NO_3_)_2_·6H_2_O] and 2-methylimidazole (mIm) ligands.^8^ The material with a sodalite cage topology is then formed by molecular self-assembly, a process where the basic building blocks—metal and linker—aggregate spontaneously to form a highly ordered extended 3D structure: the crystalline framework. While the ZIF-8 crystals may materialize as nano- or microcrystals exhibiting rounded or faceted shapes, the development of size- and shape-controlling syntheses, in turn, benefits considerably from a detailed understanding of the crystallization process.

**Figure 1:**
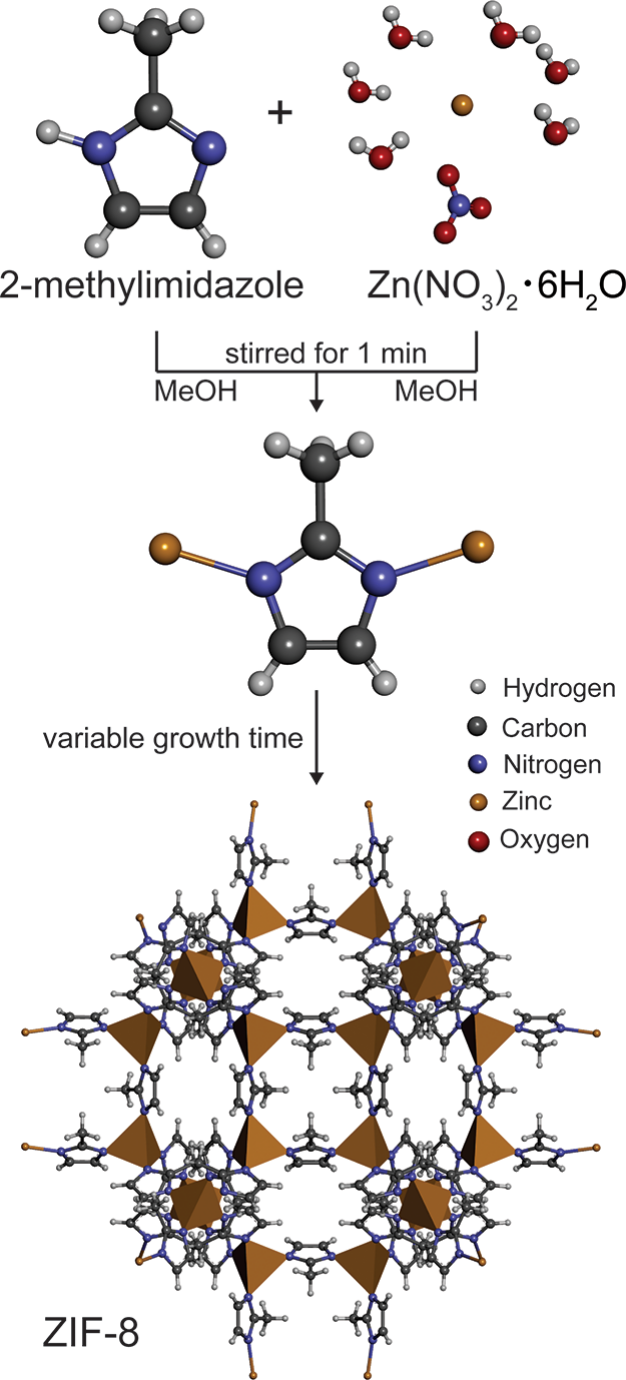
Schematic of the synthesis route of ZIF-8.

In particular, the formation of ZIF-8 crystals, or MOF crystals in general, can be divided into nucleation and growth (Figure 1).^9^ Because the nucleation is driven by random fluctuations in the bulk solution without dedicated nucleation sites, little can be done to tailor this mechanism. The crystal growth, on the contrary, can be manipulated by changing the molar ratio of reactants, amount of solvent, and other stimuli to yield crystals with the desired sizes and shapes. Indeed, several studies have scrutinized the impact of the growth time, temperature, or use of different modulators on the formation of ZIF-8 nanocrystals by employing techniques such as atomic force microscopy (AFM) or scanning electron microscopy (SEM) combined with diffraction or adsorption–desorption analysis to capture the size distribution, shape, and crystallinity of the crystals.^10−15^

Yet, if seen in the context of controlling the material properties, another factor plays a crucial role in crystalline nanomaterials: the presence of defects.^16^ Defect engineering opens pathways to locally tune the intrinsic porosity, create open metal sites, and modulate the surface properties of MOFs, which has significant implications for separation, gas capture, catalysis, and mechanical responses.^17−25^ Perhaps the model system for defective MOFs is UiO-66 (Universitetet i Oslo); here, nanoregions of ordered defects had been reported, which inspired detailed studies on the defects and their effects on the material.^19,26−29^ For instance, structural defects in UiO-66 were observed using a combination of low-dose transmission electron microscopy (TEM) and electron crystallography, further confirming the increased catalytic activity for defective structures.^30^

Little is known, in contrast, about defects in ZIF-8; this may be partly because this material is considered to be among the most stable frameworks, but while this is true, it is, in fact, very plausible that defects or other irregularities may occur during the crystal growth process, possibly with a significant impact on the performance of the materials, and its subsequent long-term stability.^31^ Hitherto, only the feasibility of local defects in ZIF-8, such as linker and metal vacancies or dangling linkers, has been predicted by computational modeling.^32,33^ However, despite that fact that a few other studies have focused on the implications of defects in gas separation^34^ and storage^35^ or found surface-terminating defects,^36^ many open questions remain. One might ask, do defects occur and transform during crystallization of ZIF-8? Also, to what extent do the defects affect the properties and, as such, the performance of the material for potential applications including gas capture, chemical sensing, and catalysis?

To answer these questions, we employ scattering-type scanning near-field optical microscopy (s-SNOM), merging AFM with IR spectroscopy to enable near-field IR nanospectroscopy. It is this combination that can unravel the fine-scale features, mechanisms, and chemical interactions of MOFs at the nanoscale by yielding a Fourier transform infrared (FTIR) spectrum of a local 20 nm spot.^37−41^ Previously, we demonstrated the capability of this technique to probe individual MOF-type nanocrystals, such as ZIF-8, opening the door for discovering their previously unreported characteristics from a new perspective: at the single-crystal level.^39^ Compared with conventional techniques, this nondestructive approach allows not only direct imaging but also simultaneous measurement of the sample’s properties including chemical information or physical properties at a resolution akin to AFM.^40^ For instance, the mechanical properties can be obtained with the same setup, albeit operated in contact mode instead of tapping mode. In these tip force microscopy (TFM) measurements, a force–distance curve is attained from every pixel of the AFM scan by retracting the AFM tip, measuring the required force to do so. The local stiffness is then determined from the force difference between the maximum force and the reference trigger point on the curve.^42^ From the measured stiffness data of a sample surface, an image of the Young’s modulus map can be derived. By simultaneous measurement of the shape, size, chemical composition, and mechanical properties at the nanoscale, using a suite of multimodal near-field techniques—s-SNOM, nanoFTIR, AFM, and TFM—allows us to shine new light on the growth process of the ZIF-8 nanocrystals.

In this study, we explore how defects transform during crystallization of ZIF-8, further elucidating their impact on the mechanical properties of the material. First, we study ZIF-8 nanocrystals before turning to microcrystals, obtained through two different synthesis routes. To examine the intermediate steps of the crystallization, the crystal growth was stopped after 1, 3, and 60 min by removing some material from the same batch and performing three washing steps. The experimental evidence was corroborated by *ab initio* density functional theory (DFT) calculations through a comparison between ZIF-8 ideal and defective model structures.

## Results and Discussion

### Inhomogeneity after a Short Growth Time

**Figure 2:**
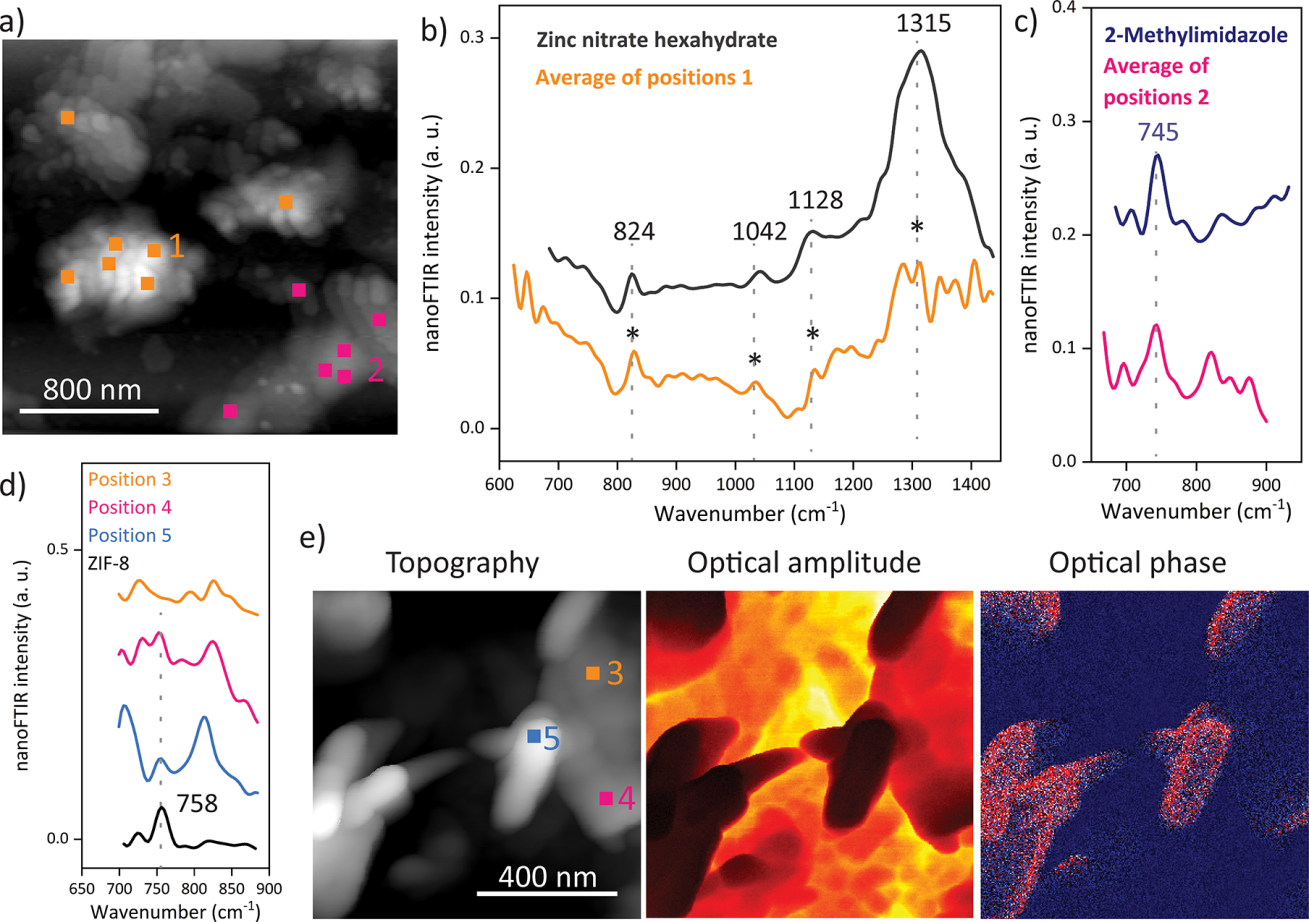
Near-field IR spectroscopy of ZIF-8 with 1 min growth time. (a) The AFM image indicates the positions where the local nanoFTIR spectra (b and c) are measured, which resemble the spectra of the reactants. The curves are shifted in the *y* direction for a better comparison, and the spectra measured on the nanocrystals show a higher noise level than that of the nanocrystals obtained from larger quantities of reactants. The emergence of a characteristic ZIF-8 peak (d) is already observable by local probing at the positions shown in the AFM image (e), and local variations are further revealed in the optical amplitude and optical phase images (e).

After 1 min of growth time, the sample is dominated by inhomogeneous regions, although small, rounded shapes are already observable at the nanoscale (Figure 2). Local probing of these nanoscopic morphologies reveals their spectral resemblance with the metal source, as confirmed by the presence of the characteristic vibrational peaks of Zn(NO_3_)_2_·6H_2_O at 824, 1042, 1128, and 1315 cm^–1^ in the nanoFTIR spectrum (Figure 2a,b). In contrast, the planate areas of the sample exhibit spectral features of the mIm linkers, such as the distinctive vibrational band at 745 cm^–1^ (Figure 2a,c). Of course, it is the brief crystallization period and the lack of successfully formed nuclei and subsequent Ostwald ripening^43^ that explain these distinct regions, where either the uncoordinated linker or the metal reactant dominates without evolved interactions between them. Only a closer examination reveals local variations, as illustrated in the optical amplitude and phase images, which qualitatively contrast materials with different optical properties (Figure 2d,e). Interestingly, the nanoFTIR spectra measured at different local spots with 20 nm resolution show contributions from both materials, even displaying the emergence of the characteristic peak of ZIF-8 at 758 cm^–1^, assigned to an out-of-plane ring mode of the framework (Figure 2d). This finding confirms not merely the growing chemical interactions and bond formations but provides a snapshot of the assembly stage of the framework itself, which, at least partially, is beginning to crystallize within the first minute. Nonetheless, the large amount of uncoordinated linker and metal salt may indicate which defects could materialize during further crystallization.

### Defects Gradually Disappearing with Prolonged Crystallization

**Figure 3:**
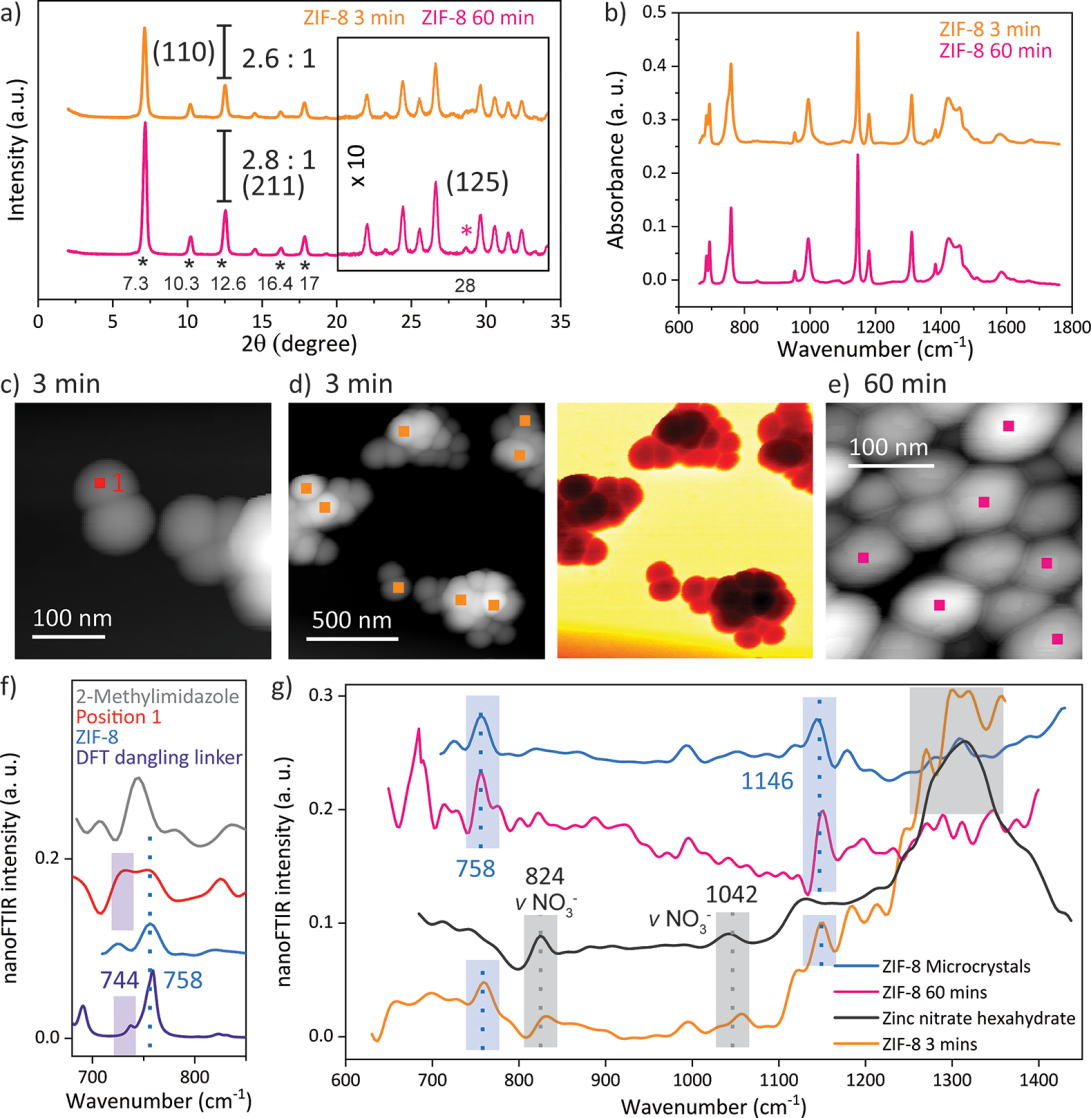
Comparison between the ZIF-8 nanocrystals obtained after 3 and 60 min: (a) The XRD pattern confirms the crystallinity of the sample obtained after 3 min. (b) The ATR-FTIR spectra reveal that ZIF-8 crystals are formed after 3 min. (c and d) The topography and optical amplitude of the nanocrystals were imaged after 3 min of growth time with s-SNOM. (e) The AFM image of the final growth stage of the ZIF-8 crystals is given. (f) The nanoFTIR spectra of a 20 nm spot, as indicated in the AFM image (c), compared with the spectrum of the linker, ZIF-8, and a simulated FTIR spectrum of ZIF-8 with a dangling linker defect are given. (g) The average of several local spectra (positions illustrated in parts d and e) reveal local variations, which vanish with prolonged crystallization.

After a growth time of only 3 min, the ZIF-8 nanocrystals have already formed, as confirmed by comparing their X-ray diffraction (XRD) pattern and attenuated-total-reflectance (ATR)-FTIR spectra with those measured on crystals after a growth time of 60 min (Figure 3a,b). In particular, the Bragg peaks from XRD are fully resolved, matching the reported characteristic peaks at 2θ values of 7.3, 10.3, 12.6, 16.4, and 17° and thus clearly revealing the crystallinity for the nanocrystals attained after 3 min (Figure S1).^8^ Here, the high intensity of the (110) peak at 7.3° is attributed to the formation of ZIF-8 with a regular rhombic dodecahedron morphology, which resembles the final stage of the growth of ZIF-8 crystals.^14^ Small variations, however, can be detected, such as the absence of the (125) peak at 28°, or a changing relative intensity of the (110):(211) planes, corresponding to the two most intense diffraction peaks. Albeit minimal, a rise in the intensity of the (110) plane, accompanied by a narrowing of the full width at half-maximum (fwhm) from 0.299 to 0.288, is observed upon prolonged crystallization (Figure S2). Either this can be attributed to the particle size effects via the Debye-Scherer equation or, given that the increase in the average size is minimal (Figure S3), this may indicate a stronger long-range ordering, thus implying a higher crystallinity.^44^ At first glance, these findings are in good agreement with the previously reported result, where the crystallinity increased with the synthesis duration due to the Ostwald ripening process.^13−15^ It has been suggested that 5 min was insufficient to grow ZIF-8 crystals,^13^ but here, paradoxically, it is the marginal increase of the crystallinity, and the ATR-FTIR spectrum of the crystals with 3 min growth time, which matches that of the final growth stage, that indicate the completion of the self-assembly of ZIF-8 nanocrystals after only 3 min.^13−15^

Although these bulk measurements, in combination with the close topological resemblance of the shape and size, suggest the growth of ZIF-8 nanocrystals after a relatively short crystallization time, near-field IR spectroscopy reveals that, in fact, these crystals are far from being defect-free. For instance, the average of several local nanoFTIR spectra measured on individual crystals exposes the strong contribution of Zn(NO_3_)_2_·6H_2_O, with its characteristic peaks around 824 and 1042 cm^–1^ associated with the stretching modes of the NO_3_^–^ anions (Figure 3d,g).^45^ As opposed to ATR-FTIR, where the spectra are obtained from an average of the bulk polycrystalline material, the nanoFTIR spectrum is measured locally with a probing depth of only a few nanometers, or tens of unit cells. Therefore, the presence of vibrational modes associated with the metal reactant indicates local variations close to the surface of the framework, such as different termination groups or undercoordinated metal clusters. One reason for the predominant appearance of these modes in the vibrational spectrum averaged over several positions could stem from the recurring defect of missing linkers, where nitrate molecules are instead coordinated to the zinc cations. The defect of missing linkers accordingly leads to a strong contribution of metal clusters or, in other words, zinc-rich regions that can emerge on the outermost surface of ZIF-8.^36,46^ Creating open metal sites through defects can be leveraged as a strategy to enhance the reactivity of framework materials, whether it is in catalytic applications or gas adsorption. Here, the undercoordinated zinc ions close to the crystal surface present additional reaction sites. Zhang et al. hypothesized that such defects could be responsible for higher adsorption energy with water for ZIF-8, and our chemical characterization can finally provide the so-far-lacking experimental proof to explain these previous findings.^47^ Precisely this feature, however, may alleviate the long-term stability of the material by deteriorating its hydrophobicity.^34^

Turning even more locally, individual point spectra show the superposition of peaks assigned to ZIF-8 (758 cm^–1^) and the mIm linkers (745 cm^–1^), further uncovering local defects associated with the linker, such as a partially coordinated linker or a dangling linker, as the termination units close to the crystal surface (Figure 3c,f). This is confirmed by DFT calculations using the *CRYSTAL* code.^48^ A defective ZIF-8 crystal was modeled, where a metal vacancy introduced dangling linker groups. While both point defects and extended defects are likely to exist in zeolites, up to now, the latter has not been identified in the sodalite-type framework topology that is characteristic for ZIF-8.^33,49^ Therefore, we concentrate solely on point defects, even though these local techniques might probe a combination of various types of defects. In the simulated FTIR spectrum, an additional peak appears at 744 cm^–1^, which is assigned to the vibrational modes of the dangling linker. Of course, such irregularities close to the external surface of the crystallites are to be expected when materials on the nanoscale are examined. However, none of these structural defects are observed in the nanoFTIR spectrum of the nanocrystals with a growth time of 60 min. Instead, the local measurements resemble the average spectrum and reveal neither local variations nor individual contributions of the reactants (Figures 3g and S4). This leads to the conclusion that structural defects, such as the different crystal lattice terminations, are gradually disappearing with prolonged crystallization.

### Mechanical Property Evolution with Vanishing Defects

**Figure 4:**
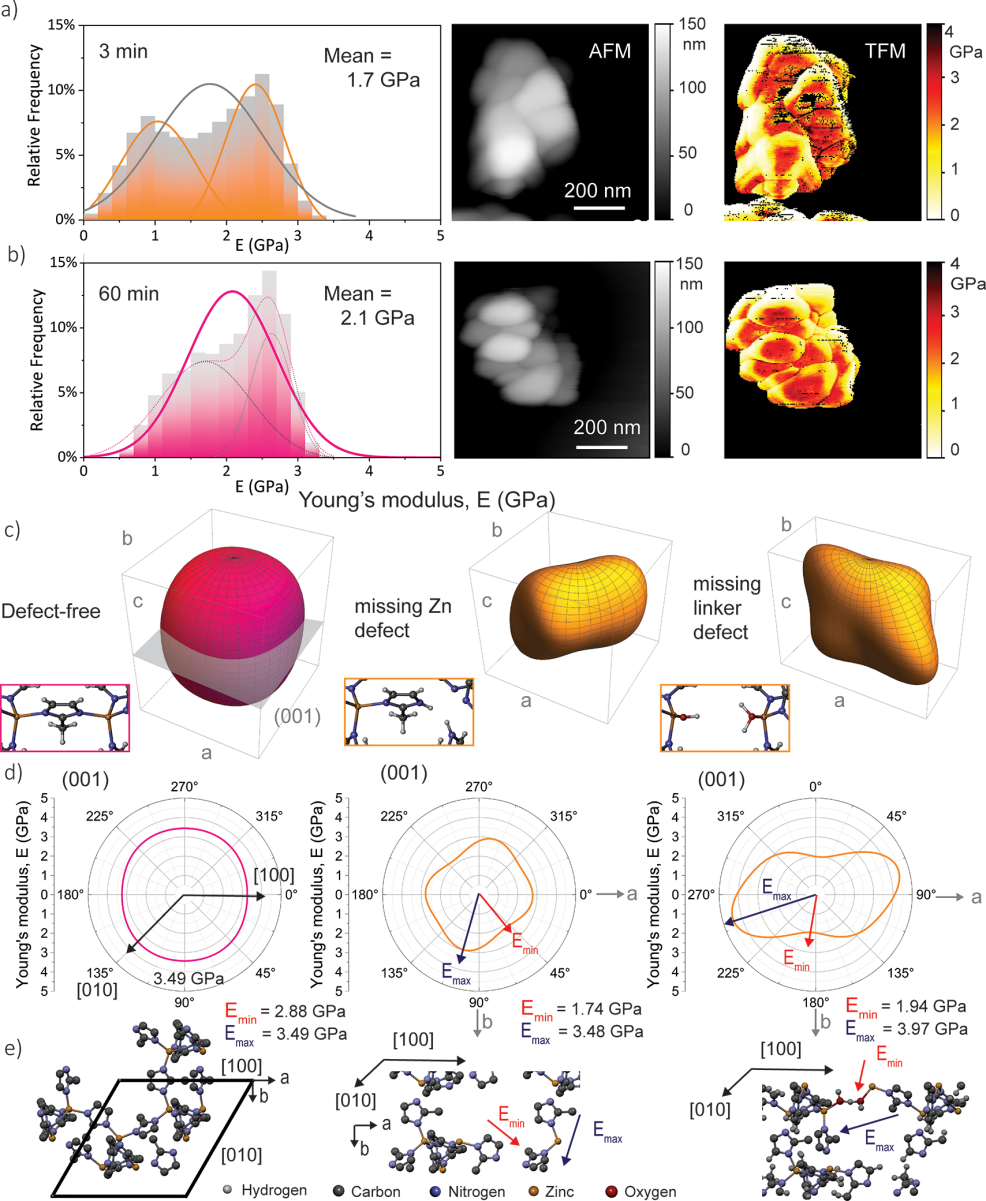
TFM on the ZIF-8 nanocrystals with different growth times (top, 3 min; bottom, 60 min). (a and b) Histogram and normal distribution of the Young’s modulus corresponding to data collected on the nanocrystals on each pixel, along with AFM images, and mapping of the Young’s modulus, measured with a tip force. (c) Young’s modulus representation surface in 3D spherical coordinates, along with schematics of ZIF-8 and defective structures. (d) Polar plots projected onto the (001) planes. (e) Schematics of the structure–property relationships, indicating the orientations of the maximum and minimum Young’s moduli.

Of course, the different termination units, the undercoordinated metal, and the dangling linker all invite the question of how and to what extent they might influence the properties of the nanocrystals, which are so intrinsically linked with the framework assemblage, arrangements of the functional groups, and characteristics of the pore. Even if only slightly, defects may affect the material’s stability by disrupting the crystalline order of the framework material, but whether the relationship between the defects and their impact on the material performance can be precisely established remains to be studied. Because of the lack of other experimental techniques to probe the mechanical properties of individual nanocrystals, this is best done with TFM, where the Young’s modulus *E* (i.e., ratio of the uniaxial stress over the strain in the elastic regime) is measured at every pixel of the scan, while simultaneously imaging the topography at nanoscale resolution. In that way, the local stiffness of the sample under investigation is acquired with a resolution akin to AFM imaging (Figure 4).^42^ It is worth mentioning that the artifacts in the measurements (e.g., black lines) have been filtered out before the mean stiffness of the nanocrystals is derived (Figure 4a,b). The stiffness has a generally higher variance than that previously measured for ZIF-8 with conventional techniques, whether it is with indentation or AFM nanoindentation, an observation that can be attributed to the effect of edges. Here, establishing contact between the AFM tip and the sample is challenging, which leads to weaker interactions and thus lower stiffness, accordingly reducing the mean stiffness. Another drawback of such a local surface technique is that, in order to generate 3D Young’s moduli from the experimental data, probing the sample from all angles would be required, which is unfeasible for individual nanocrystals like those prepared for AFM-based measurements. Yet, at such small scales—as measured with TFM—the obtained stiffness reflects the local mechanical response of individual nanocrystals.^50^ Given the same calibration, these TFM measurements can thus be employed for a comparison between different materials. For ZIF-8 crystals with a growth time of 3 min, the Young’s modulus is attained from one scan with a mean and standard deviation value of *E*(3 min) = 1.7 ± 0.7 GPa (or between 0.8 and 3.4 GPa considering nine different scans), which is lower compared with the stiffness of the crystals at the final growth stage when it was determined that *E*(60 min) = 2.1 ± 0.5 GPa (or 1.4–4.6 GPa for six scans; Figure S7). Likewise, the crystals with short crystallization unveil more variance in the local Young’s moduli, even revealing a bimodal distribution (Figures 4a and S6). A simple way of putting this is to say that higher crystallinity, and thus stiffness, is achieved with prolonged crystallization, but there is more to it than that, especially because the increase in crystallinity is marginal, as discussed earlier. An alternative explanation might be to suggest that the observed structural defects, whether they are different termination units, uncoordinated metal clusters, or dangling linkers, introduce local disturbance to the otherwise periodic bulk structure and thus disrupt the stability of the framework as a whole; in other words, one might describe them as local disorder with a far-reaching impact. This applies particularly to the mechanical properties due to their dependencies upon the long-range order of the crystal. The observed phenomenon that the local structural stiffness of the framework, or the mechanical stability of crystal in general, is lowered with increasing defect level coincides with previously reported simulations, where the elastic constants were calculated for defective UiO-66 crystals; however, the assumption that defects in ZIF structures lead to different mechanical properties demands further proof.^51^

Hence, we performed DFT calculations with the *CRYSTAL17* code^48^ to compute the elastic tensors *C*_*ij*_ for three different structures of ZIF-8: first, the ideal defect-free crystal was simulated using the PBEsol0-3c method.^52^ Derived from this ideal unit cell, the missing zinc defect was introduced as a zinc vacancy and by the replacement of two N–Zn bonds with N–H bonds, thereby creating dangling or undercoordinated linkers. The third system, which, in turn, simulates the missing linker defect, was attained by removing a linker group, while an associating water and the conjugate base of the proton-donating group filled the two unsaturated metal sites. Visualizing the elastic representation surfaces (Figure 4c) and the associated mechanical properties derived from the *C*_*ij*_ tensors (Tables S2 and S3 and Figures S8–S11) reveals the differences in the mechanical properties due to defects, corroborating the trends derived from the experimental findings. As shown in Figure 4c,d, the Young’s modulus of the defect-free crystal is highly isotropic, consistent with previous studies on the elastic constants of a ZIF-8 single crystal.^53,54^ On the contrary, with the introduction of a missing zinc defect, an anisotropic behavior is revealed. The anisotropic mechanical response of ZIF-8 has previously been investigated, e.g., as a function of the pressure,^54^ but in this work, we focus on the impact of point defects on the mechanical properties.

In the case of missing zinc defects, although the maximum stiffness in the direction of the stable, undisturbed zinc bonds is akin to that of the perfect crystal, the minimum Young’s modulus is significantly smaller (Figure 4e). If forces are applied in the direction of the longest pore width, the dangling linker groups can now rotate or twist because of the missing zinc defect, which decreases the framework stiffness accordingly. Similarly, the effect of increased anisotropy due to defects can be noticed in the model of the missing linker; in fact, it is this system where it becomes most evident. Here, a bond between the associated water and conjugate base is formed to yield a mechanically stable system. This, however, changes the conformation of the pore significantly: in the direction of this additional bond, the framework stiffness is increased because of the shorter bond lengths and associated pore conformation. As a consequence, the maximum Young’s modulus is even larger than that in the defect-free case. Yet, in the vertical direction, these weak hydrogen bonds are easily disrupted under compressive or tensile forces, leading to a much larger pore than that in the defect-free crystal, which describes the decrease in the minimum Young’s modulus.

The theoretical results above can explain the observed characteristics of the local stiffness measured with TFM: the average stiffness and, as such, the material’s stability are generally reduced in the defective structures. If inverted, the effect is that the structural flexibility, adsorption, or other anisotropic responses to external stimuli can be enhanced in an otherwise “rigid” MOF (greater stiffness) by introducing structural defects.^51^ On the question of anisotropy, it is worth mentioning that anisotropy is linked to the distribution of defects along given directions. The calculations assume a periodic occurrence of the introduced defects, while, in reality, the number of defects and their orientation is, most likely, random. Yet, even if only one defective site is modeled per unit cell to preserve a mechanically stable system, the defect is replicated throughout the entire crystalline structure because of periodic boundary conditions. Therefore, the DFT models translate to the single-crystal level when the elastic constants are computed, enabling us to make the link to experiments. However, although the simulations predict the mechanical properties of the defective ZIF-8 systems under investigation and the local measurements confirm the corresponding decrease and anisotropy in stiffness, it remains challenging to distinguish between the type or location of defects from these nanoscale measurement techniques.

### Defect Transformation in Microcrystals

**Figure 5:**
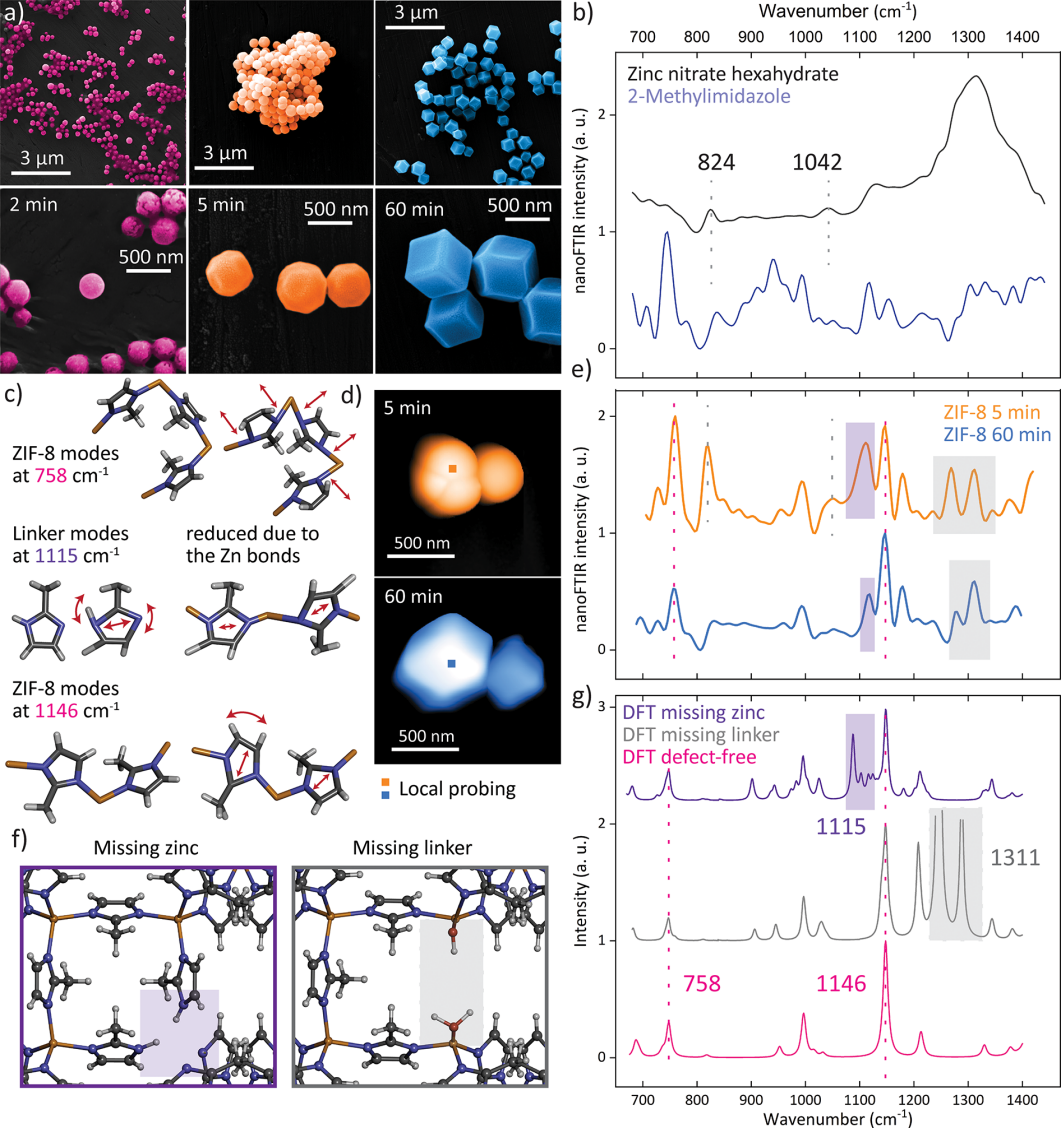
Transformation of ZIF-8 microcrystals. (a) SEM imaging of ZIF-8 crystals with different growth times. (b) nanoFTIR spectra of the reactants. (c) Schematic representations of key vibrational modes. (d) AFM images of ZIF-8 crystals obtained after 5 and 60 min, indicating crystals selected for local probing. (e) Corresponding local nanoFTIR spectra obtained from the averaging of 20 point spectra on the crystal. (f) Schematic representations of the simulated defects. (g) Corresponding simulated FTIR spectra compared with a perfect ZIF-8 crystal.

Additional observations are made by tracking the changes in key vibrational bands during the crystallization of ZIF-8 microcrystals, which further reveals how defects transform. As shown in the SEM images in Figure 5a, the crystal shape evidently changes from the round morphology obtained after 2 min of growth time to exhibition of the first facets after 5 min, eventually reaching the rhombic dodecahedron shape at the final growth stage after 60 min. This is accompanied by a gradual increase in the averaged crystal size from 200 to 500 nm. A comparison of the two nanoFTIR spectra, averaged for the 5 and 60 min growth times, with the DFT calculations depicts a close resemblance of the samples yet unravelling several salient changes in the peak positions and intensities (Figure 5d,e,g; see Figure S5 for a complete scan of the crystals). First, the nanoFTIR spectrum corresponding to the longer growth process shows a better match with the calculated spectrum of an idealized, periodic crystal because, with prolonged crystallization, the number of ideal bonds increases, and thus the positions and intensities resemble the calculated IR spectrum. Second, a detailed study of the individual vibrations, considering the DFT calculations as well as the nanoFTIR spectra of the reactants before synthesis, gives insights into both the structural and chemical changes happening during crystallization of the 3D framework. The pronounced peak at 758 cm^–1^, for instance, correlates with the symmetric out-of-plane bending of the mIm ring and associated motions of the H–C=C–H bond present in the framework structure (Figure 5c). Conversely, the peak at 824 cm^–1^ is related to the vibrations of the metal salt [Zn(NO_3_)_2_·6H_2_O], which is not expected to appear after the construction of ZIF-8, where both reactants are used up entirely to form ZIF-8 crystals. However, because the vibrational mode associated with the NO_3_^–^ stretching is still present in the nanoFTIR spectrum, the crystals formed after only 5 min still contain residuals of excess reactants, thus suggesting that some metal clusters are not fully coordinated with the mIm linkers.

Given that extensive washing steps have been performed for all samples, one can conclude that the metal cations are, albeit poorly coordinated, bound to the framework. There is some doubt where the metal clusters could precisely be located, but the strong appearance of the vibrational mode, in fact, indicates a repeating pattern of this defect close to the crystal surface; most likely, it is the defect-terminating zinc clusters with missing linkers that could further explain why the crystals, rather than resembling the stable rhombic dodecahedron shape, only slightly imply facets after a 5 min growth time. In comparison with the characteristic peak of ZIF-8 at 758 cm^–1^, the vibrational mode associated with the metal reactant at 824 cm^–1^ depicts a reduced relative intensity from 0.74:1 to 0.48:1 with a longer crystallization time, a finding that already contains, in essence, everything that has been observed for the nanocrystals. With prolonged growth time, the defects of ill-terminating metal clusters that are bound to molecules of the reactant gradually disappear. The same phenomenon is revealed in the reduction of the less pronounced peak at 1042 cm^–1^; likewise, this peak simultaneously vanishes as the complete framework is assembled (Figure 5b,e).

So far, the emphasis fell on the defects attributed to the undercoordinated metal clusters; however, it is further evident that the defect of the dangling linker (or missing zinc) can be detected at such local scales. In particular, the vibrational mode at 1115 cm^–1^, a peak associated with asymmetric in-plane ring stretching of the uncoordinated linker C–N–H bonds, is strongly present in the early stage of the crystal growth process. Interestingly, these modes appear in the simulated spectrum of a defective ZIF-8 crystal, where dangling linkers have been introduced by virtue of a metal vacancy. Meanwhile, in the final stage of crystal growth, this mode has reduced in intensity because the N–H bonds in the linker are replaced by the stable N–Zn bonds suppressing this vibration (Figure 5c).

Thus, in the 5 min crystallization, the high relative intensity of this vibration with 0.85:1 compared with the ZIF-8 peak at 1146 cm^–1^—the latter is attributed to the C–H rocking and ring stretching in the framework—reveals the defect of unsaturated, ill-terminating ligands, or so-called dangling linkers, which are only bound to one zinc atom. As a result, the free-space vibrations of the linker, particularly the mode associated with the unwieldy asymmetric ring stretching and the N–H bonds, are enhanced and thus detectable in the nanoFTIR spectrum. Once bound to two zinc atoms in a fully assembled framework, these motions are mostly constrained, as indicated by the disappearance of this mode, or the decrease in the relative intensity to 0.47:1, with prolonged crystallization. In fact, the DFT assessment shows that this asymmetric ring stretching mode is even more suppressed in a defect-free crystal, as implied by the low relative intensity between the two simulated peaks (0.05:1). This suggests that, to some extent, this type of defect is found to prevail even when the final stage of the microcrystal growth is reached after 60 min. One can thus conclude that ordered dangling linker defects can exist in ZIF-8 microcrystals, including those deemed seemingly perfect.

Thus, the potential of measuring defects in ZIF-8, which is not by any means confined to the initial stages of early crystallization, emerged. The significance of this finding for understanding the prototypical “stable” ZIF-8 crystal is so great that it is worth discussing a little further. Whereas the bulk ATR-FTIR measurements perfectly match the calculated FTIR spectrum (Figure S12), the local nanoFTIR spectra do not. Instead, it is possible to pinpoint local characteristics in the vibrational modes and, in combination with computational modeling, associate them with chemical and structural peculiarities at the nanoscale. A glance at the vibrations between 1250 and 1350 cm^–1^ will further illustrate this because these additional vibrations are observed in the local spectra of ZIF-8 crystals and can be assigned to missing linker defects, yet they appear neither in the ATR-FTIR measurement nor in the calculated crystal, which is defect-free (Figure 5e,g). However, this is precisely what the nanocrystals are not because they feature these modes, namely, the missing linker defects, at such local scales. Akin to the DFT calculations, where the resulting two unsaturated metal sites are filled with an associating water and the conjugate base of the proton-donating group, the associated vibrational modes appear in the nanoFTIR spectrum, indicating the presence of such an aqueous linker vacancy at the crystal surface. These results highlight that the defect sites may alleviate the hydrophobicity, and as such the long-term stability of ZIF-8, by exposing open metal sites to solvents, gases, or other reactants. On the other hand, the same phenomenon could also enhance the adsorption capabilities of the material—what had previously been regarded as structural defects or, at best, imperfections may come to be considered as merits. For applications of defect engineering in ZIF-8, we refer to the findings of Lee et al.,^34^ Cheng and Hu,^35^ and Tian et al.,^36^ who have shown an increase in the performance of defective ZIF-8 in gas separation and storage.

## Conclusions

In this work, we tracked the transformation of defects in ZIF-8 by locally probing nano- and microcrystals at different stages during crystal growth with s-SNOM. As opposed to established techniques, which measure a spatial average over the bulk material, the use of s-SNOM yields chemical information with a nanoscale resolution akin to AFM imaging. In that way, the coexistence of defects can be observed during crystallization by pinpointing the vibrational dynamics of a 20 nm spot, encompassing several unit cells. Whereas after a 1 min growth time large amounts of uncoordinated linker and metal reactants are still dominant, it is already possible to note the presence of round nanocrystals; here, local probing with nanoFTIR confirms the emergence of characteristic IR peaks of ZIF-8, thus suggesting that the framework is already forming after such a short crystallization time. Indeed, ZIF-8 nanocrystals that match the characteristics of the final growth stage are attained after only 3 min, as corroborated with conventional characterization techniques like XRD, ATR-FTIR, and AFM. However, there is one distinction, which can only be revealed with nanospectroscopy: that between ZIF-8 crystals with perfect periodicity and those with structural defects. For instance, the local nanoFTIR spectra show the dominance of zinc ions, or of ill-coordinated metal clusters, close to the surface of the nanocrystals with 3 min growth time, even revealing the coexistence of vibrational modes associated with ZIF-8 and the uncoordinated linker at the same 20 nm spot, thereby indicating defect-terminating ligands. With prolonged crystallization, or the ripening of the nanocrystals, these defects gradually vanish, and the final growth stage is reached after 60 min. Perhaps the most significant implication of this defect evolution—it is even more striking than observing the defect itself—consists of a change of the mechanical properties. A glance at the local Young’s modulus, as measured with nanoscale resolution using TFM, has illustrated this: not only is the structural stiffness significantly lower in the presence of defects, but it also exhibits a higher local anisotropy. These experimental findings are confirmed by DFT calculations, where the mechanical properties of ZIF-8 and its defective structures have been computed. These trends are attributed to the fact that defects introduce local disorder to the otherwise highly ordered 3D periodic framework, ultimately compromising the material stability, although their impact on the material properties might extend even further. Knowledge of how defects affect the nanoscale mechanical behavior of ZIF-8 is key to targeted applications in catalysis, sensing, and gas capture, where not only is mechanical stability required but also defect engineering could further improve the material’s performance.

The same phenomenon of defect transformation during crystallization was observed in ZIF-8 microcrystals. While the individual crystals transform from a spherical morphology to the rhombic dodecahedron shape, the chemical changes that underpin these features were tracked on the single-crystal level for the first time. After a growth time of 5 min, the faceted shape begins to emerge, yet several local structural defects are determined with nanospectroscopy; those include undercoordinated metal clusters and dangling linkers. Again, the trend of defect evolution is epitomized in their gradual disappearance with prolonged crystal growth time, although the defect of dangling linkers, if only slightly, is deemed to prevail. Similarly, defect sites of missing linkers lead to unsaturated metal sites, which adsorb water molecules, as confirmed with nanospectroscopy and DFT calculations. This phenomenon of open metal sites is crucial for the application of ZIF-8 because such additional adsorption sites allow for enhanced catalytic behavior, targeted chemical sensing, or increased gas capture. Out of that understanding grows doubt as to whether the stable, faceted ZIF-8 crystals, which have typically been assumed to be essentially defect-free, are, in fact, perfect or whether the defects were just invisible to most of the characterization techniques employed to date.

Hence, this first use of s-SNOM to probe defects in individual crystals offers an alternative, nondestructive method to low-dose TEM and electron crystallography for studies on defects in MOFs, with the additional advantage of providing chemical information at such local scales (∼20 nm). This novel tool can thus spark the exploration of local defects, not only in MOFs but also in other crystalline nanomaterials. In particular, the combination of s-SNOM with theoretical modeling of the mechanical properties unveils the missing link between previous studies on ZIF-8 that either computationally showed the feasibility of defects or employed defect tuning for enhancement of the material’s performance. In addition, the first application of TFM in the field of MOFs enables the link to be established between the physical, chemical, and mechanical properties to shed new light on the implications of defects or, in fact, any features at the nanoscale. We envisage that these findings and techniques invite future studies on defects in MOFs and cognate framework materials either to evaluate the stability of “stable” MOFs for targeted application or, on the contrary, to leverage local defect engineering to tailor the material performance.

## Experimental Section

### Synthesis of ZIF-8 Nanocrystals

ZIF-8 nanocrystals were synthesized by dissolving 4.5 mmol of zinc nitrate hexahydrate [Zn(NO_3_)_2_·6H_2_O; 98%, Sigma-Aldrich] and 13.5 mmol of 2-methylimidazole (mIm; 98%, Sigma-Aldrich) in 60 mL of methanol, respectively. After the two clear solutions were combined, the white colloidal solution was rigorously stirred for 1 min and then left to form the nanocrystals. Immediately, some material was removed, diluted in fresh methanol, and washed three times. Each washing step encompassed centrifugation at 8000 rpm for 5 min, followed by solvent exchange with fresh methanol, and sonication for 30 s. The same procedure was repeated after 3, 5, and 60 min: after these time intervals, some material was removed and thoroughly washed to stop the growth process.

### Synthesis of ZIF-8 Microcrystals

Two precursor solutions were prepared by dissolving 4 mmol of Zn(NO_3_)_2_·6H_2_O and 4 mmol of mIm in 40 mL of methanol, respectively. A mixture was obtained by combining the two precursor solutions, which was stirred for 1 min and then left to stand. After specific time intervals (2, 5, and 60 min), some material was removed from the batch and immediately washed three times with methanol and centrifugation.

### Sample Preparation for Nanoscale Analytics

Each sample was diluted in methanol and drop-casted onto a silicon substrate. To eliminate any excess solvent, the sample was dried in a vacuum oven at 80 °C for at least 30 min. The spectra of the reactants were measured by dissolving Zn(NO_3_)_2_·6H_2_O (98%, Sigma-Aldrich) or mIm (98%, Sigma-Aldrich) in methanol, respectively. Likewise, the solution was drop-casted onto a clean silicon substrate and dried at 80 °C for 30 min.

### Powder X-ray Diffraction (PXRD)

The PXRD patterns were measured at a step size of 0.02° and step speed of 0.01°/min using a Rigaku MiniFlex diffractometer equipped with a Cu Kα source and validated against the simulated XRD pattern (CSD database code: VELVOY).

### ATR-FTIR

ATR-FTIR measurements on bulk material were performed using a ThermoFisher Scientific Nicolet iS10 FTIR spectrometer with a spectral resolution of 4 cm^–1^.

### SEM Imaging

SEM images of the samples were obtained with a TESCAN LYRA3 electron microscope. Backscattered-electron and secondary-electron SEM images were obtained at 10 keV under high vacuum. The false-color images were produced using Adobe Photoshop.

### nanoFTIR

The near-field optical measurements were performed with a neaSNOM instrument (neaspec GmbH) based on tapping-mode AFM, where the platinum-coated tip (NanoAndMore GmbH; cantilever resonance frequency 250 kHz; nominal tip radius ∼20 nm) was illuminated by a broadband femtosecond laser. The coherent mid-IR light was generated through the nonlinear difference–frequency combination of two beams from fiber lasers (TOPTICA Photonics Inc.) in a GaSe crystal. Laser A was selected for measurements covering the range from 700 to 1400 cm^–1^. Demodulation of the optical signal at higher harmonics of the tip resonance frequency eliminated background contributions to yield the near-field signal, comprising the amplitude and phase of the scattered wave from the tip. When a pseudoheterodyne interferometric detection module was employed, the complex optical response of the material was measured, where the real part refers to the nanoFTIR reflectance and the imaginary part depicts the nanoFTIR absorption spectrum. Each spectrum was acquired from an average of 14 Fourier-processed interferograms with 10 cm^–1^ spectral resolution, 2048 points per interferogram, and 14 ms integration time per pixel. The sample spectrum was normalized to a reference spectrum measured on the silicon substrate. All measurements were carried out under ambient conditions.

### TFM

TFM was employed as an additional module to the neaSNOM instrument, but here AFM was operated in contact mode. The z-piezo driver was modulated at a sinusoidal motion with an amplitude of 40 mV and a modulation frequency of 610 Hz. A complete force–distance cycle was performed at this rate for each pixel with an integration time of 33 ms (200 × 200 pixels per image). The technique followed the description of a pulsed-force mode.^42^ Each cycle comprised an approach of the AFM tip from free oscillation until the establishment of contact with the sample, followed by subsequent retraction. More specifically, contact was established because of the (negative) attractive force between the tip and sample surface. Once in contact, the piezo drove the tip even closer to the sample until the (positive) repulsive force reached a maximum. Upon retraction of the tip, the repulsive force decreased and was replaced by the attractive force because of the adhesion between the sample and tip until contact was lost and the tip freely oscillated. From this cycle, various properties could be derived.^42,55,56^ For instance, the topography image was obtained from the maximum force, which was fed back to the control circuit to maintain a constant normal force. An adhesion image could be created based on the maximum adhesion force for each pixel. The local stiffness was attained from the force difference between the maximum force and a set point in the repulsive part of the force signal. Hard surfaces led to a larger force difference than that observed for soft surfaces. Calibration measurements were carried out on the silicon substrate, and a polystyrene/poly(methyl methacrylate) polymer blend sample with known Young’s moduli was used to validate the calibration. The mean local stiffness was obtained from the stiffness images by filtering out outliers and the background region containing the substrate. The normal and bimodal distributions were then calculated with the integrated analysis tools in *OriginPro 2019*.

## Computational Details

### DFT Calculations

Theoretical vibrational spectra along with elastic constants of ZIF-8 and defective ZIF-8 models were calculated with the PBEsol-3c method, a cost-effective 'composite method' developed for solid-state calculations.^52,57^ It is based on a hybrid Hartree–Fock/DFT Hamiltonian combined with a double-ζ basis set, augmented with a semiclassical dispersion term and a geometrical counterpoise correction, which provides a good trade-off between cost and accuracy.^58,59^ The calculations were carried out with a development version of the periodic *ab initioCRYSTAL17* code running in MPP mode on ARCUS-B, part of the high-performance computing facility at the University of Oxford (Oxford, U.K.), and on the U.K. national HPC facility ARCHER2.^48^ The missing metal (or so-called dangling linker) defect was created by removing a metal atom and replacing two N–Zn bonds with N–H bonds similar to the uncoordinated linker. Removing a linker group, in turn, led to the missing linker defect, where the two unsaturated metal sites were filled by an associating water and the conjugate base of the proton-donating group. After geometry optimization, vibrational frequencies at the Γ point were computed, and the Berry phase approach was employed to calculate the IR intensities.^60,61^ Subsequently, a simulated spectrum was obtained by fitting the calculated IR intensities with Lorentzian peak shapes with a fwhm of 10 cm^–1^. To improve the match with the experimental data, the calculated IR spectra were scaled using distinct scaling factors for different spectral ranges, unlike adopting an overall scaling constant.^62^ For the range from 600 to 850 cm^–1^, the IR spectrum was scaled with a factor of 0.936, while the region between 850 and 1050 cm^–1^ was scaled by 0.964. Higher wavenumbers were scaled by a factor of 0.958.

The single-crystal elastic constants of the elasticity matrix (tensor) were calculated using the numerical first derivative of the analytic cell gradients.^63^ These values correspond to the independent elastic stiffness coefficients, *C*_*ij*_.^53^ The unique coefficients were obtained via deformation of the optimized structure, using a three-point formula, in the symmetrically required directions of both positive and negative amplitudes. These deformations correspond to tensile and compressive strains required to obtain the elastic response. The magnitude of each individual strain deformation is defined as 1%, ensuring that the response is in the purely elastic region. For visualization of the elastic tensors and calculation of the mechanical properties, the *Elate*,^64^*Elam*,^65^ and *Mathematica*(66) softwares were used to generate the 3D representation surfaces of different elastic moduli. Descriptions of the individual mechanical properties are given by Tan et al.^67^

## Supporting Information Available

Additional details about material characterization, including XRD, AFM, nanoFTIR, TFM, ATR-FTIR, and SEM, as well as DFT computational details and results (PDF)
