# Supplementary material for: Defect Engineering in Metal–Organic Framework Nanocrystals: Implications for Mechanical Properties and Performance
Source: ACS Appl Nano Mater. 2022 Mar 8;5(5):6398–409. doi: 10.1021/acsanm.2c00493 (PMC9150067; doi:10.1021/acsanm.2c00493)
Supplement: Supplementary file 1 — an2c00493_si_001.pdf [file an2c00493_si_001.pdf]

Supporting Information

Defect Engineering in Metal-Organic Framework Nanocrystals: Implications for Mechanical Properties and Performance

Annika F. Möslein[1], Lorenzo Donà[2], Bartolomeo Civalleri[2] and Jin-Chong Tan[1]*

[1]Multifunctional Materials & Composites (MMC) Laboratory

Department of Engineering Science, University of Oxford

Parks Road, Oxford OX1 3PJ, U.K.

[2] Dipartimento di Chimica, Università di Torino,

Via P. Giuria 5, 10125 Torino, Italy

*jin-chong.tan@eng.ox.ac.uk

1. Material Characterisation 2

2.1 X-Ray Diffraction 2

2.2 AFM 5

2.3 NanoFTIR 6

2.4 Tip force microscopy (TFM) 8

2.4 Elastic stability criteria of ZIF-8 and defective structures 12

2.5 Mechanical properties of ZIF-8 and its defective structures 14

2.6 ATR-FTIR 19

2.7 SEM imaging 20

References 21

1. Material Characterisation

2.1 X-Ray Diffraction

The XRD pattern of the ZIF-8 nanocrystals with a growth time of 3 min and 60 min, respectively, are validated against the simulated pattern to confirm the formation of ZIF-8 (Fig. S1). A detailed analysis of the most pronounced diffraction peaks at $2\theta = 7.3^{\circ}$ (110) and 12.5° (211) is shown in Figure S2. With a prolonged crystallization, a minor narrowing of the full width at half maximum (FWHM) is observed.

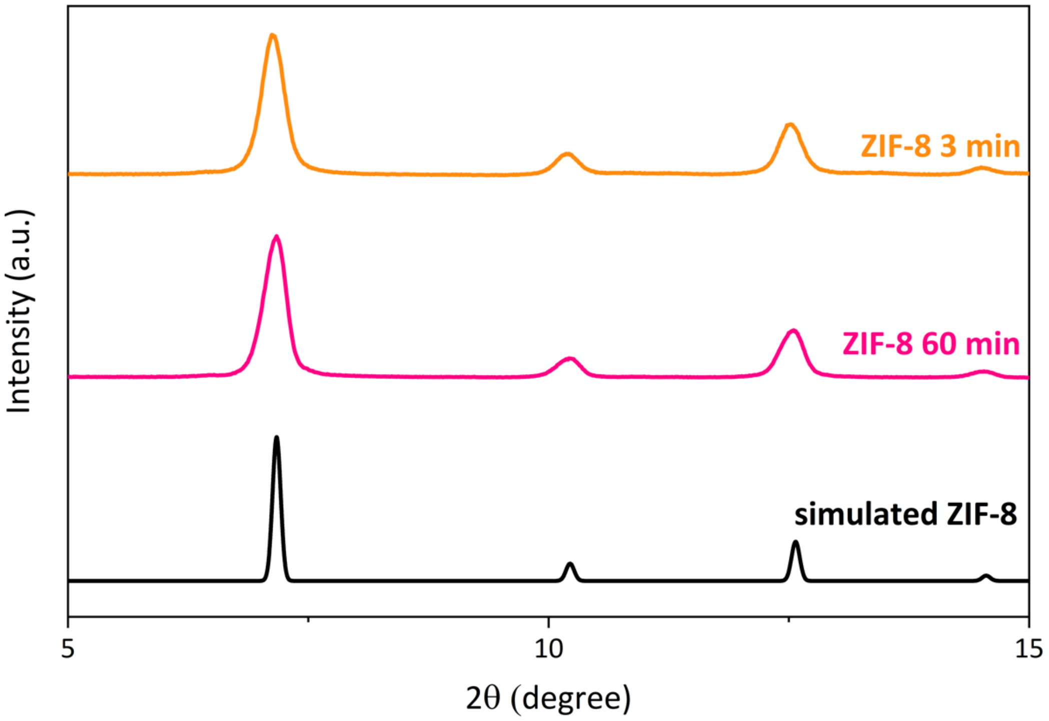

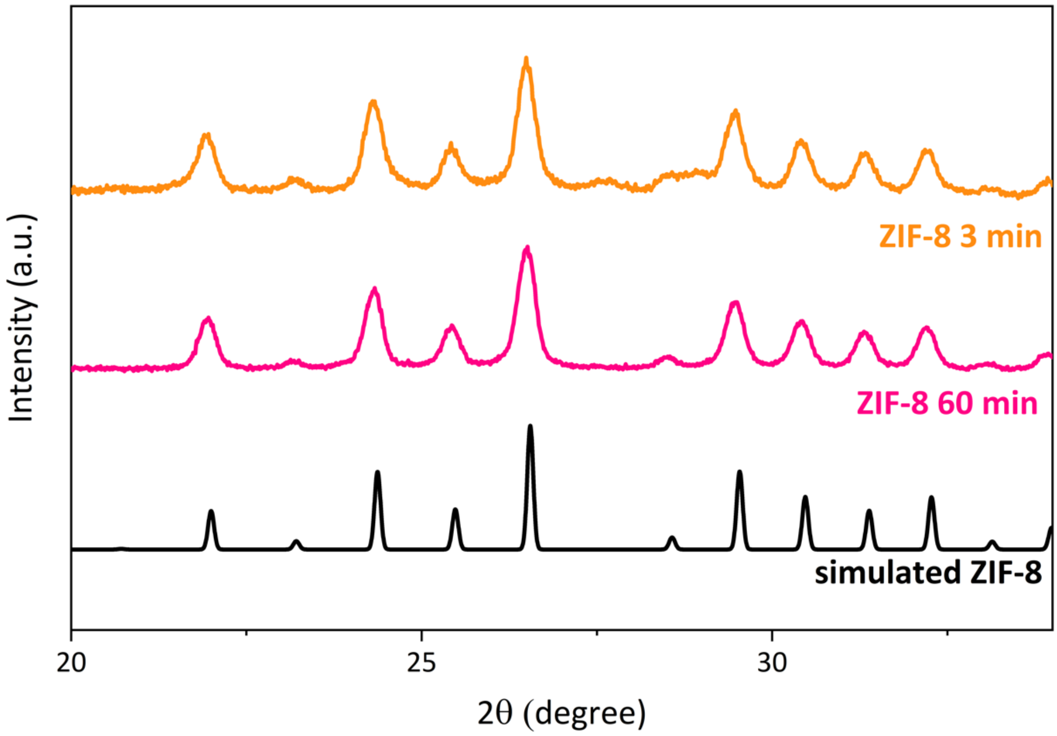

Figure S1. XRD pattern of ZIF-8 nanocrystals with different growth times compared with the simulated pattern.

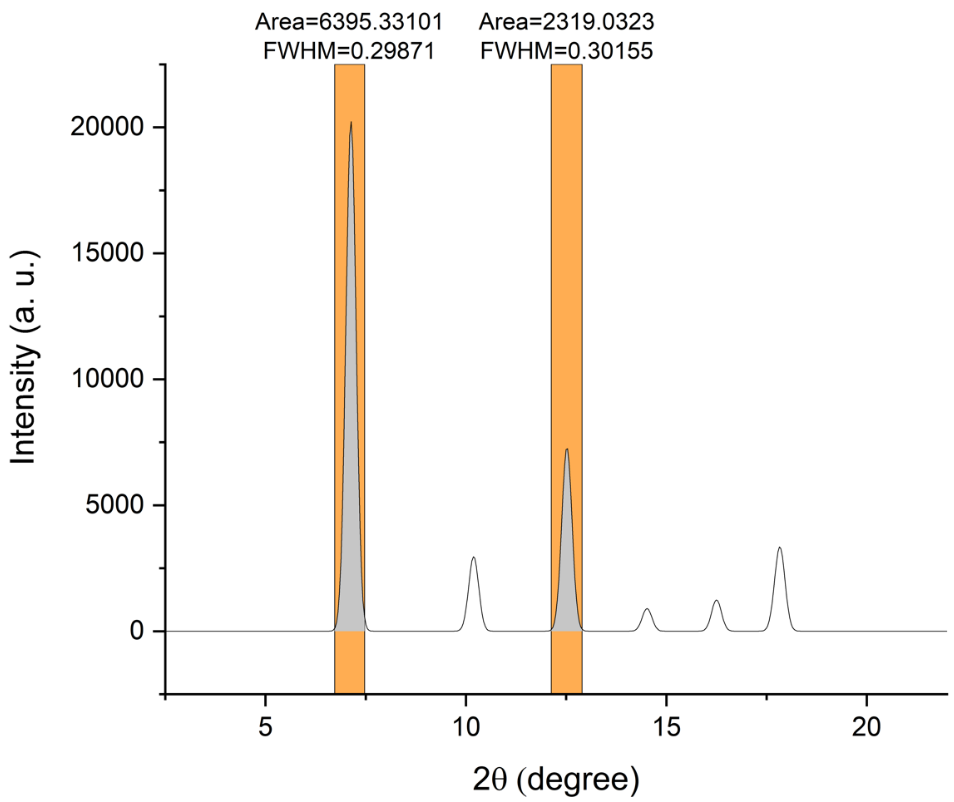

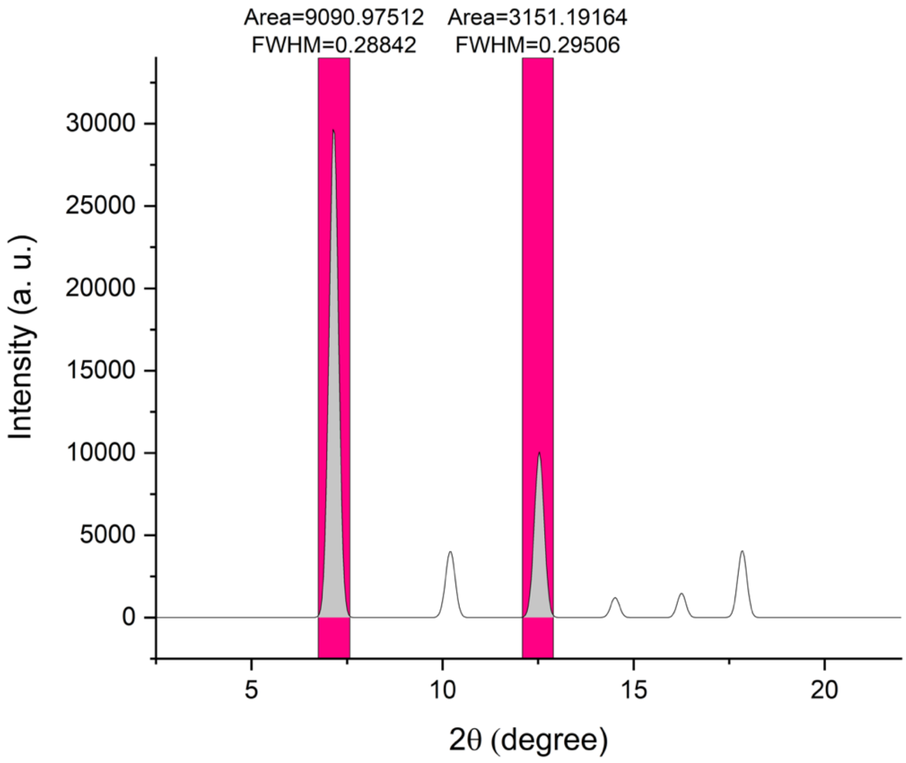

Figure S2. Peak analysis for the XRD pattern of ZIF-8 nanocrystals with a growth time of 3 mins (top) and 60 mins (bottom), respectively.

2.2 AFM

The size distribution of the nanocrystals with different growth times are compared in Figure S3.

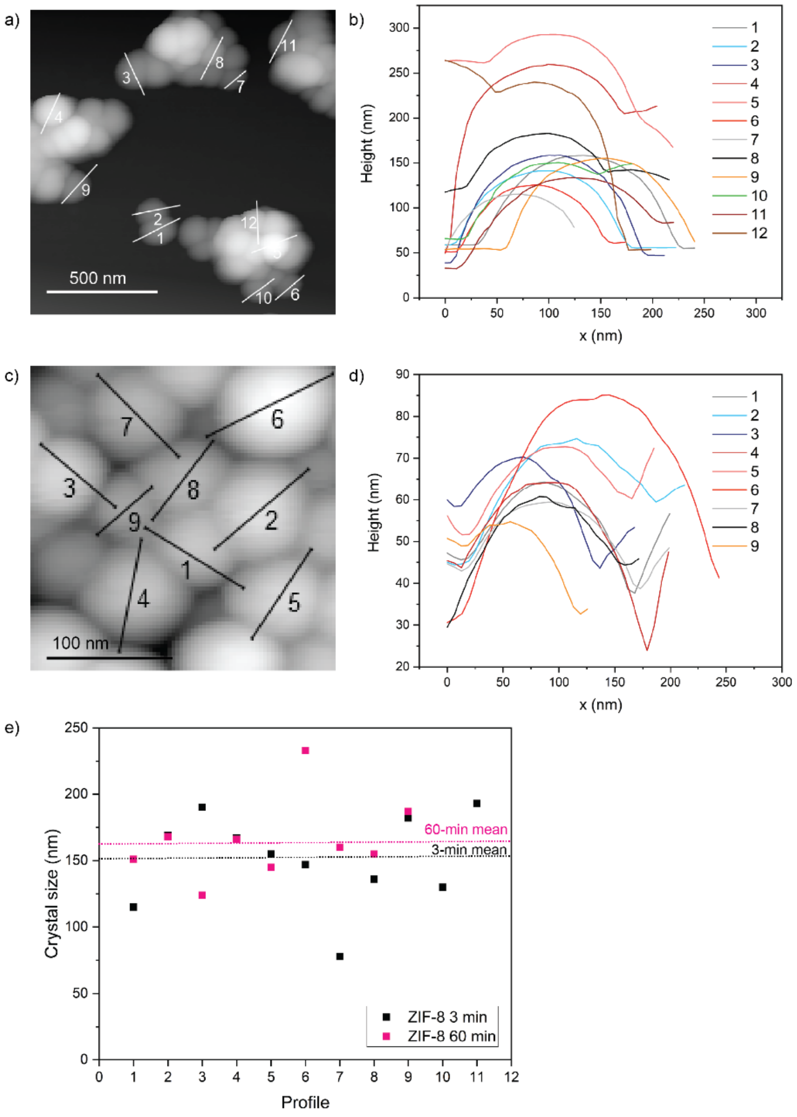

Figure S3: Size of crystals with different growth time: a) AFM image of ZIF-8 nanocrystals with a 3-minute growth time. b) Corresponding height profiles. c) AFM image of ZIF-8 nanocrystals with 60-minute growth time. d) Corresponding height profiles. e) Crystal size (width) calculated from the x-values of each profile in b) and d).

2.3 NanoFTIR

Figure S4 demonstrates how homogeneous the chemical composition of the ZIF-8 nanocrystals with a growth time of 60 minutes is. Local IR vibrational spectra indicate close resemblance with the average spectrum, calculated from various point spectra.

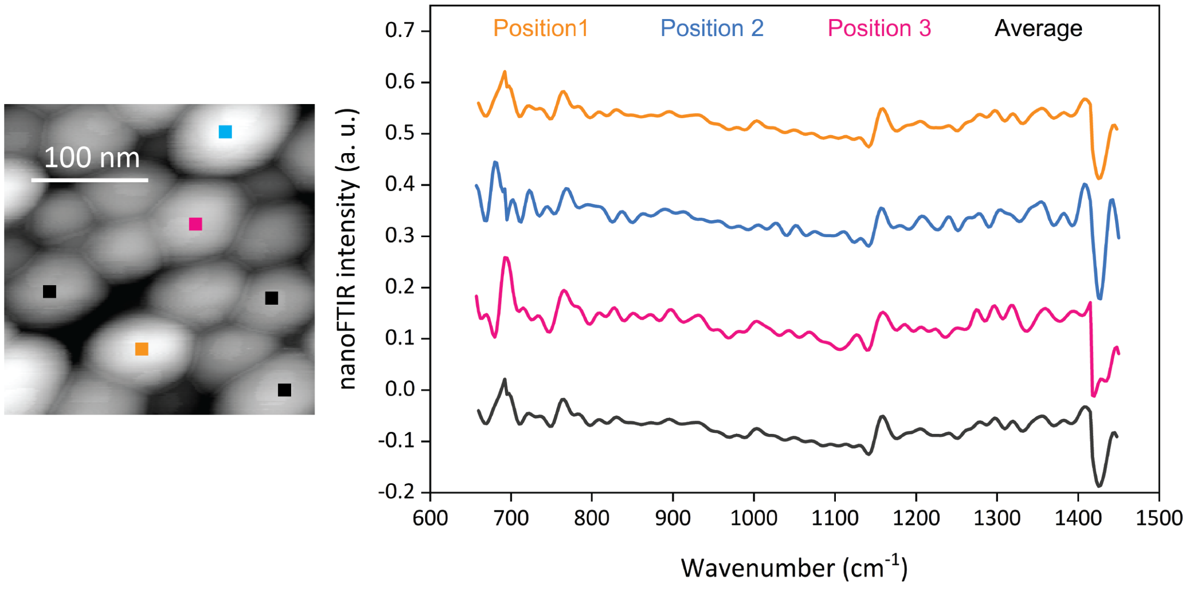

Figure S4. Local probing of the ZIF-8 nanocrystals obtained after 60 minutes yields similar chemical information.

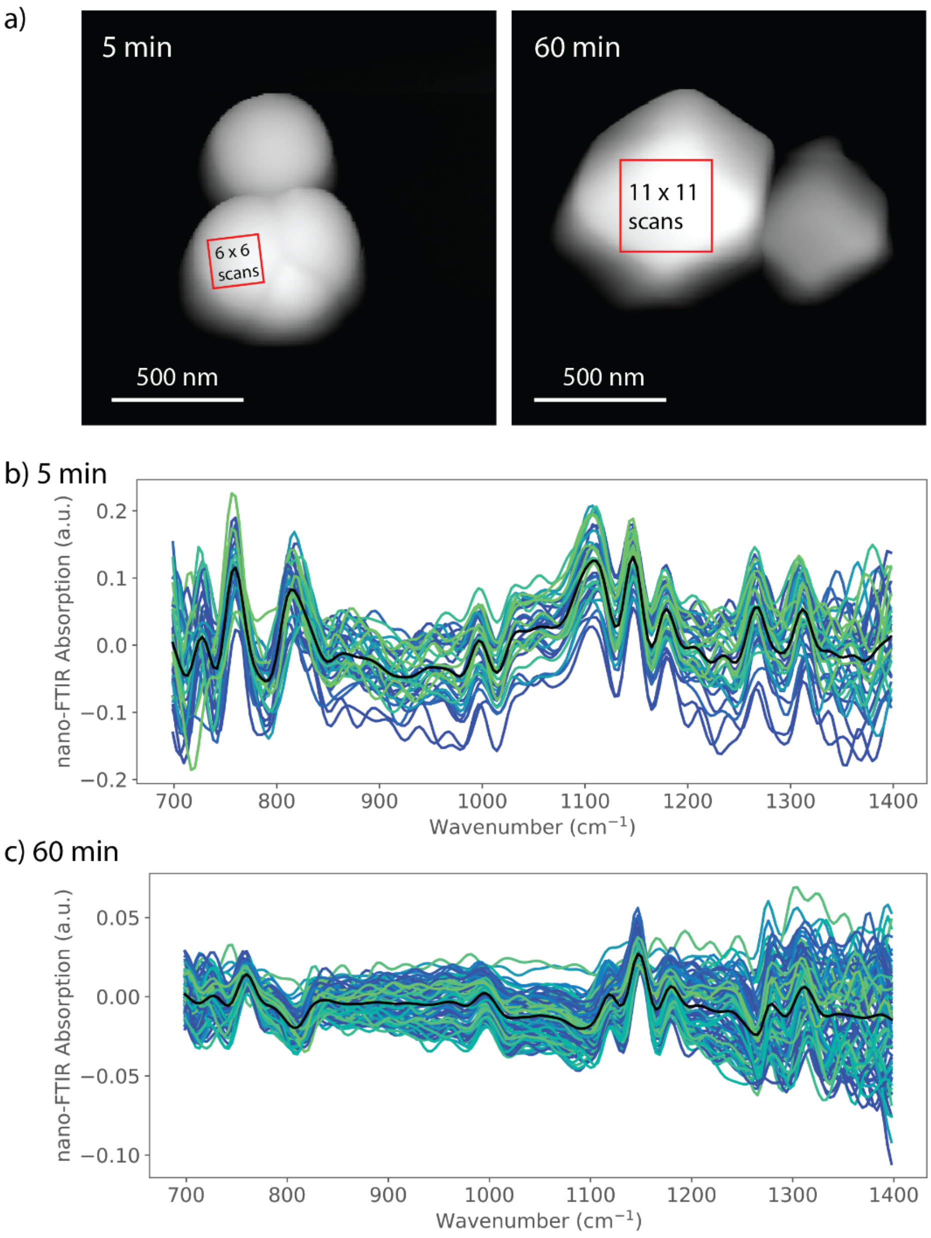

Figure S5: Spectral scanning of the ZIF-8 microcrystals with different growth time. a) AFM images of ZIF-8 crystals obtained after 5 minutes (left) and 60 minutes (right), indicating the scanned area. b) 36 nanoFTIR spectra measured from each pixel of the scanned area and their average (black line). c) 121 nanoFTIR spectra measured from each pixel of the scanned area and their average (black line)

2.4 Tip force microscopy (TFM)

Several scans were measured with tip force microscopy for every sample. For the ZIF-8 crystals obtained after a growth time of 3 minutes, the mean values for the Young's modulus *E* range between 0.8 and 3.4 GPa, while, for the sample with prolonged crystallization, the mean value lies between 1.4 and 4.6 GPa (Fig. S6, S7, Table S1). Not only is the mean significantly lower for the crystals with shorter growth time, but the variance, in turn, is also increased, and a bimodal distribution of the Young's modulus can be observed. A similar fitting on the Young's modulus measured on the 60-minutes sample further confirms the gradual transformation of defects with prolonged crystallization: comparing the first peak fit for both samples shows that the respective mean increases from a value lower than 1 GPa to more than 1.5 GPa (Fig. S6 b). In general, and apart from effects attributed to the crystal edges, the minimum Young's modulus is expected in direction of the freely rotating linker groups due to missing zinc defects. Their gradual disappearance with a longer growth time results in a higher, more isotropic stiffness, as indicated by an increased overlap of the bimodal curves towards a normal distribution (Fig. 4b, Fig. S6 b). However, even if not revealing two peaks, the curve still shows strong asymmetry; in fact, there is a strong contribution of higher values (second peak fit). These could be assigned to the maximum Young's modulus predicted for the case of missing linker defects (E_{max} = 3.97 GPa), which is larger than the more isotropic stiffness in a defect-free crystal (E_{max} = 3.49 GPa), as mentioned previously. Perhaps, while missing zinc defects gradually vanish with extended crystal growth, the zinc cations – undercoordinated because of missing linkers – attract the permanent adsorption of nitrate or water molecules.

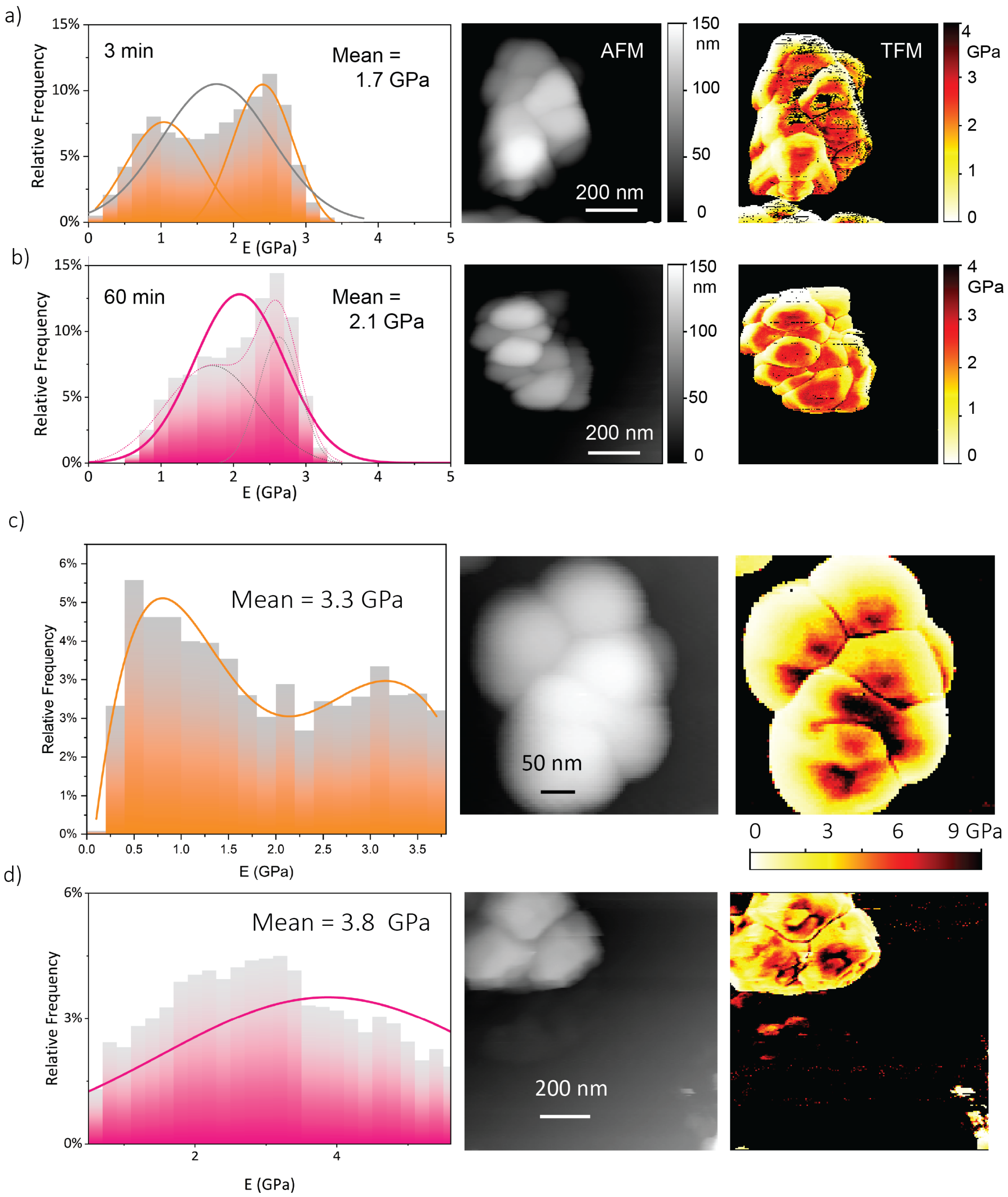

Figure S6. Tip Force Microscopy on ZIF-8 nanocrystals with different growth time. a,c) Histogram and normal distribution of Young's modulus corresponding to data collected on the ZIF-8 nanocrystals with a 3-minutes growth time, along with AFM images, and mapping of the Young's modulus, measured with tip force. b,d) Histogram and normal distribution of Young's modulus corresponding to data collected on the ZIF-8 nanocrystals with a 60-minutes growth time, along with AFM images, and mapping of the Young's modulus, measured with tip force.

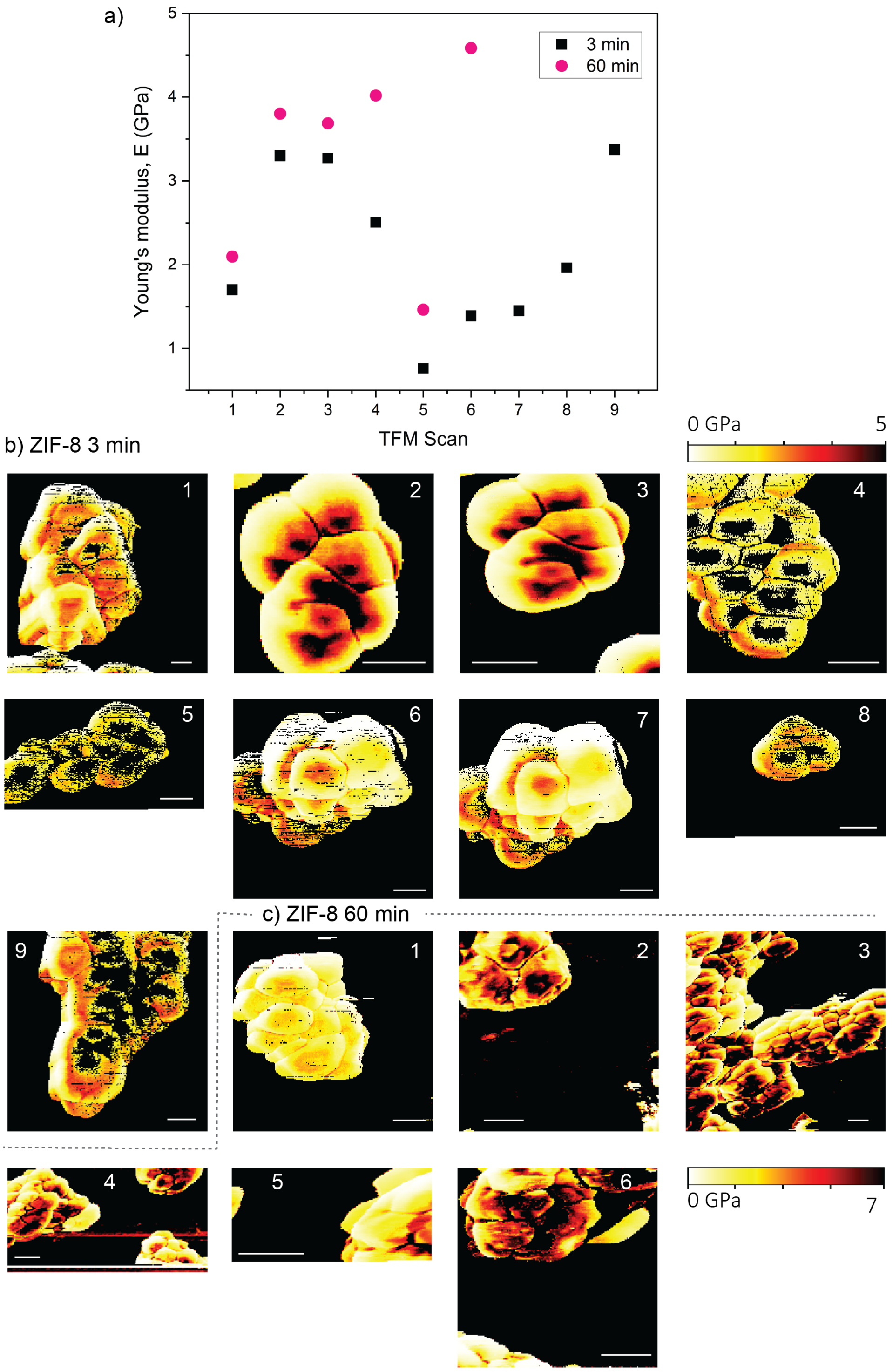

Figure S7. TFM on ZIF-8 nanocrystals. a) Mean Young's modulus *E* plotted for each TFM image. b) Young's modulus measured by TFM on ZIF-8 nanocrystals with a growth time of 3 minutes. c) Young's modulus measured by TFM on ZIF-8 nanocrystals with a growth time of 60 minutes.

Table S1. Number of pixels (points on the crystals) from which the mean Young's modulus is derived.

# Scan	**ZIF-8 3 min**		**ZIF-8 60 min**	
	N total	Mean (GPa)	*N* total	Mean (GPa)
1	8372	1.70	9000	2.10
2	5893	3.30	20837	3.80
3	5822	3.27	6734	3.69
4	14306	2.51	28490	4.02
5	24321	0.76	29952	1.46
6	17409	1.39	10777	4.59
7	15606	1.45		
8	16793	1.96		
9	19284	3.37		

2.4 Elastic stability criteria of ZIF-8 and defective structures

ZIF-8 has been simulated as an orthorhombic system after geometry optimization. As the cubic system led to negative frequencies in the frequency calculations, some symmetry restrictions had to be removed to yield a stable, orthorhombic system.

$$Elastic\ constants\ (\mathrm{GPa}) = \begin{pmatrix} 11.578 & 9.732 & 9.835 & 0 & 0 & 0 \\ 9.732 & 12.008 & 9.806 & 0 & 0 & 0 \\ 9.835 & 9.806 & 12.405 & 0 & 0 & 0 \\ 0 & 0 & 0 & 1.251 & 0 & 0 \\ 0 & 0 & 0 & 0 & 1.192 & 0 \\ 0 & 0 & 0 & 0 & 0 & 1.115 \end{pmatrix}$$

Pseudo-cubic average values: $C_{11} = 11.997,\ \ C_{22} = 1.186,\ \ C_{33} = 9.791$

Elastic stability conditions for orthorhombic systems:

$$C_{11} > 0 \ :\ C_{11} = 11.578,\ \ ok$$

$$C_{11}C_{22} > C_{12}^2 \ :\ 139.029 > 94.712,\ ok$$

$$C_{11}C_{22}C_{33} + 2\,C_{12}C_{13}C_{23} - C_{11}{C_{23}}^2 - C_{22}{C_{13}}^2 - C_{33}{C_{12}}^2 > 0 :\ \ 152.084,\ ok$$

$$C_{44} > 0 \ :\ C_{44} = 1.251,\ \ ok$$

$$C_{55} > 0 \ :\ C_{55} = 1.192,\ \ ok$$

$$C_{66} > 0 \ :\ C_{66} = 1.115,\ \ ok$$

$Eigenvalues\ of\ the\ stiffness\ matrix$ (GPa) : 1.115, 1.192, 1.251, 2.0013, 2.4061, 31.584

All eigenvalues are positive.

According to the analysis above, the ZIF-8 structure satisfies the elastic stability criteria so it is mechanically stable.[1,2]

For defective systems belonging to the triclinic Laue's class simple stability conditions are difficult to be determined. Therefore, the positivity of all eigenvalues can be considered a sufficient condition.

For the missing linker defect:

$Eigenvalues\ of\ the\ stiffness\ matrix\ (GPa)$: 0.891, 1.126, 1.395, 1.870, 2.730, 26.536

For the missing zinc defect:

$Eigenvalues\ of\ the\ stiffness\ matrix\ (GPa)$: 0.760, 0.829, 1.106, 1.871, 1.952, 20.433

According to the positive eigenvalues of the stiffness matrix, the two defective ZIF-8 structures are determined to be mechanically stable.

2.5 Mechanical properties of ZIF-8 and its defective structures

For the visualization of the elastic tensors, and the calculation of the mechanical properties, the ELATE[3], EIAM[4], and Mathematica[5] software were used. Descriptions of the individual properties are given by Tan *et al*.[6]

Table S2: Mechanical properties of ZIF-8 and defective structures.

Elastic properties	ZIF-8:	defect-free	missing Zn	missing linker
Young's modulus, E (GPa)	E_{max}	3.49	3.48	3.97
	E_{min}	2.88	1.74	1.94
	$A_E = E_{max}/E_{min}$	1.21	2.00	2.04
Shear modulus, G (GPa)	G_{max}	1.03	0.75	0.84
	G_{min}	1.25	1.11	1.54
	$A_G = G_{max}/G_{min}$	1.22	1.48	1.83
Poisson's ratio, ν (−)	ν_{max}	0.37	0.25	0.19
	ν_{min}	0.53	0.61	0.80

Table S3: Isotropic Voigt–Reuss–Hill (VRH) averaged elastic properties, corresponding to the polycrystalline state. The bulk (*K*), Young's (*E*), and shear (*G*) moduli are in GPa and the Poisson's ratio (ν) is dimensionless.

Property	K_{VRH}	E_{VRH}	G_{VRH}	ν_{VRH}
defect-free	10.52	1.15	3.33	0.45
missing Zn	5.60	0.95	2.70	0.42
missing linker	7.01	1.18	3.36	0.42

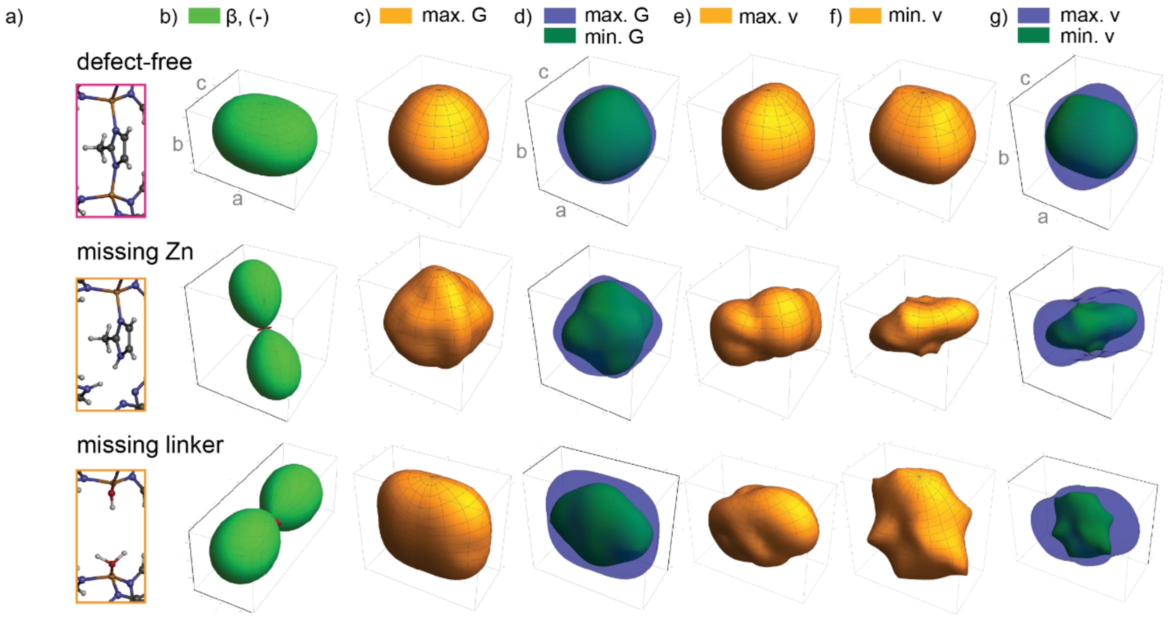

Figure S8: a) schematic of ZIF-8 and defective structures. b) Surface representation of the linear compressibility (*β*). c, d) Surface representation of the maximum and minimum shear modulus (*G*). e-g) Surface representation of the maximum and minimum Poisson's ratio (ν).

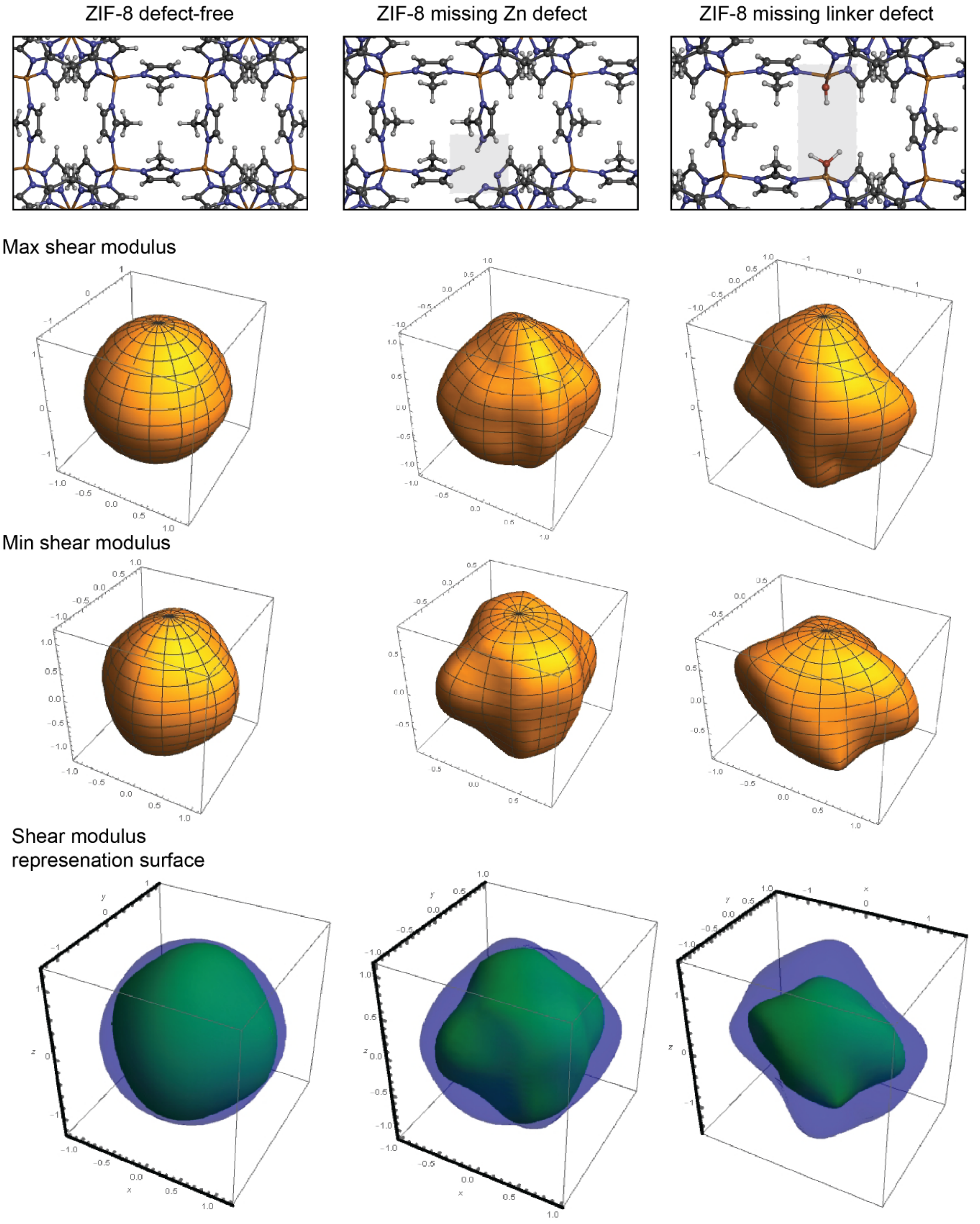

Figure S9: Shear modulus *G* representation surfaces. The blue and green surfaces denote the maximum and minimum shear moduli, respectively.

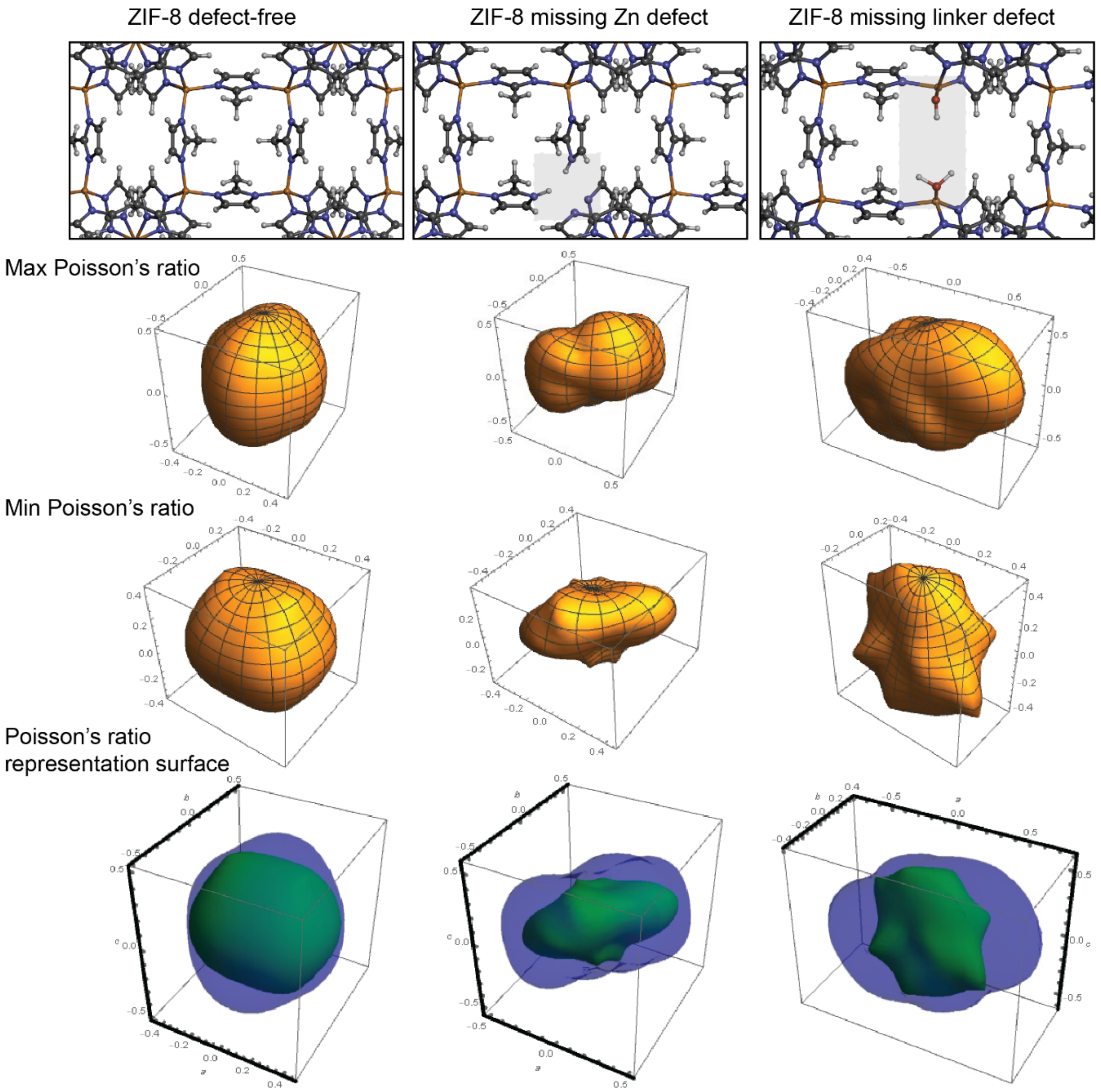

Figure S10: Poisson's ratio representation surface, where the blue surface signifies the maximum Poisson's ratio and green surface denotes the minimum Poisson's ratios.

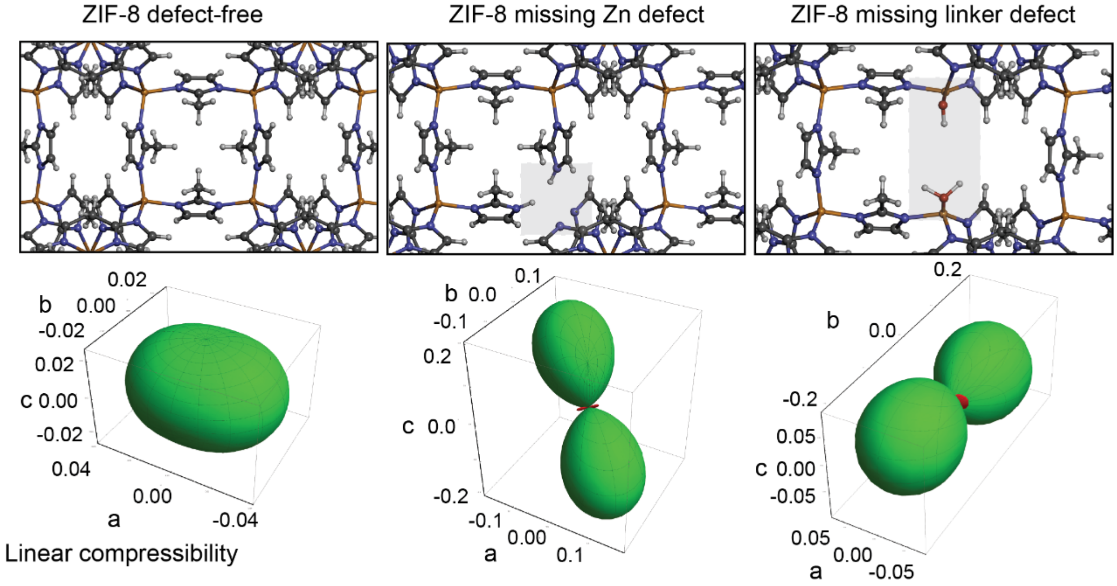

Figure S11: Surface representation of the linear compressibility β.

2.6 ATR-FTIR

Figure S12 juxtaposes the calculated IR spectrum and the ATR-FTIR spectrum measured on the polycrystalline sample of ZIF-8 microcrystals obtained after a growth time of 60 min. This shows the close match between the theoretical DFT spectrum and the experimental spectrum of a bulk sample.

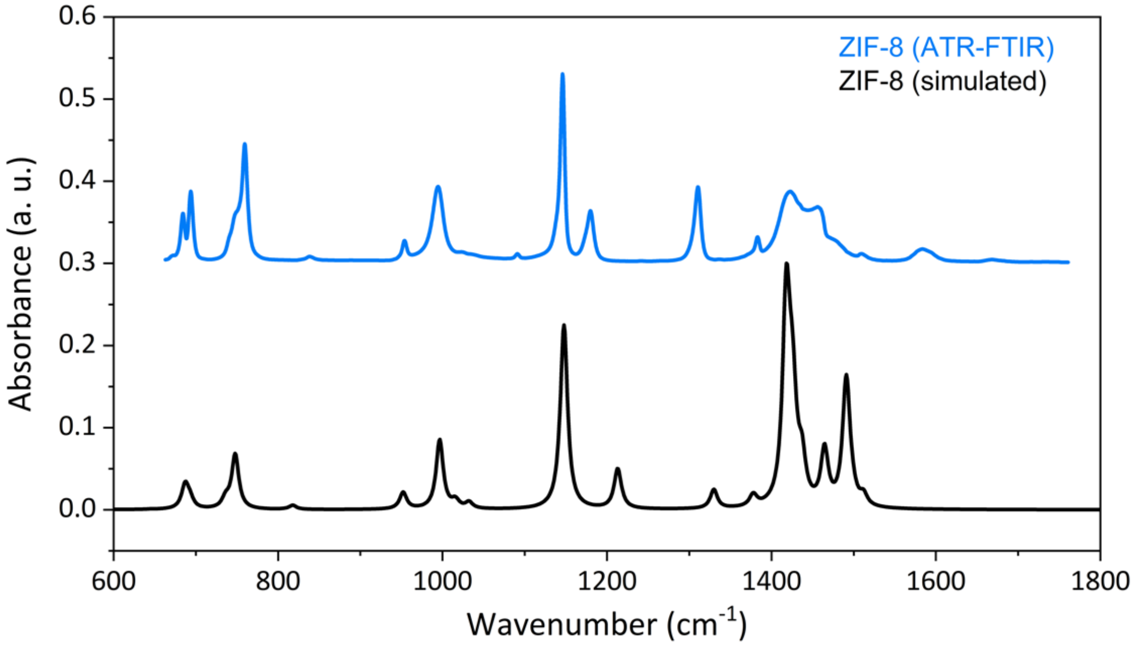

Figure S12. Comparison between the ATR-FTIR measurement of ZIF-8 bulk (polycrystalline) powder and the simulated spectrum from DFT.

2.7 SEM imaging

Additional SEM images prior to artificial colouring are shown in Figure S13.

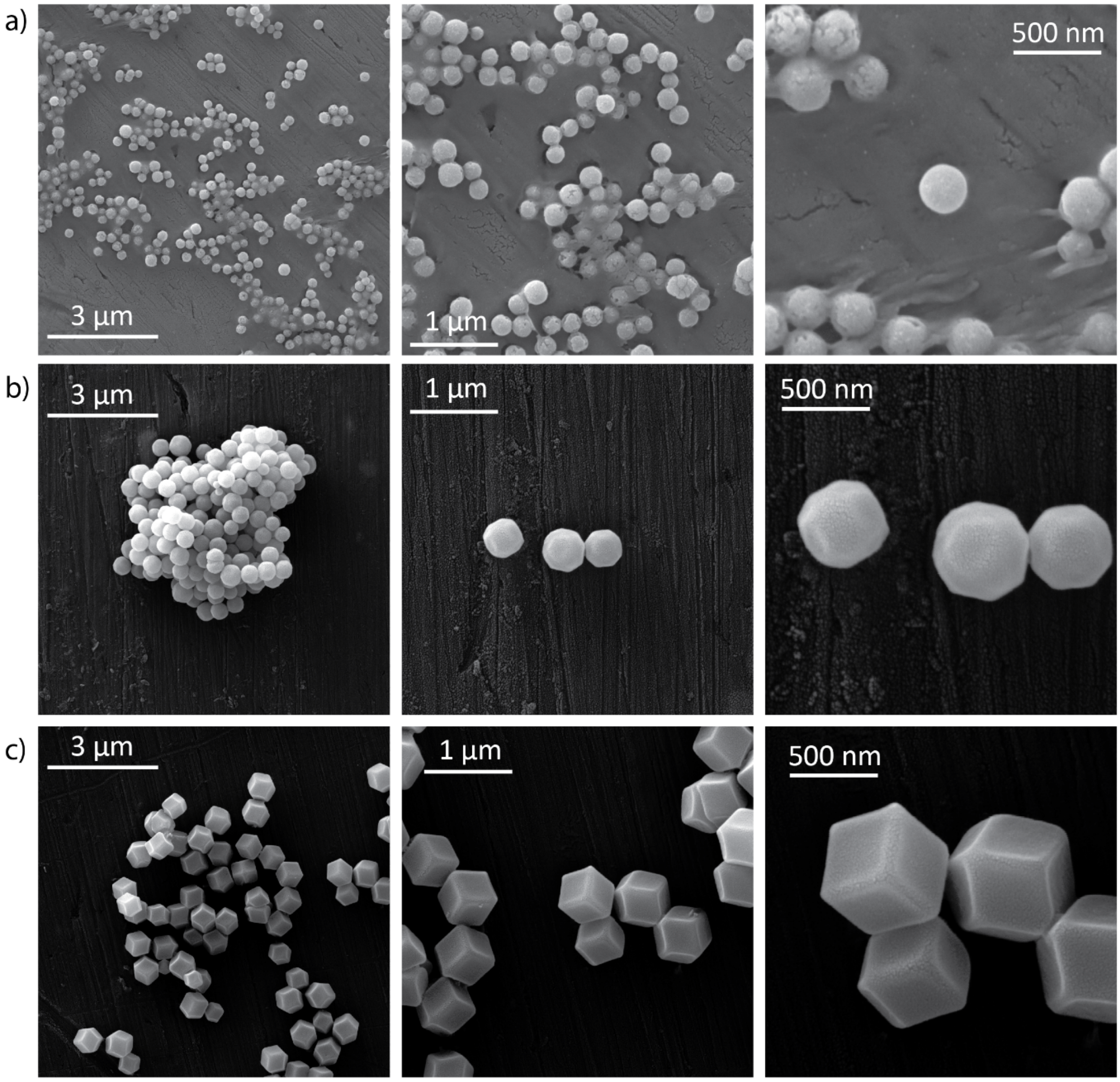

Figure S13. SEM imaging of the ZIF-8 microcrystals with different growth times: a) 2 min, b) 5 min, c) 60 min.

References

(1) Haussühl, S., *Physical Properties of Crystals: An Introduction*. WILEY-VHC Verlag GmbH & Co. KGaA: 2007.

(2) Mouhat, F.; Coudert, F.-X. Necessary and Sufficient Elastic Stability Conditions in Various Crystal Systems. *Phys. Rev. B* **2014,** *90*, 224104

(3) Gaillac, R.; Pullumbi, P.; Coudert, F. X. Elate: An Open-Source Online Application for Analysis and Visualization of Elastic Tensors. *J. Phys. Condens. Matter* **2016,** *28*, 275201.

(4) Marmier, A.; Lethbridge, Z. A. D.; Walton, R. I.; Smith, C. W.; Parker, S. C.; Evans, K. E. Elam: A Computer Program for the Analysis and Representation of Anisotropic Elastic Properties. *Comput. Phys. Commun.* **2010,** *181*, 2102-2115.

(5) *Mathematica*, Wolfram Research Inc.: 2015.

(6) Tan, J.-C.; Civalleri, B.; Lin, C.-C.; Valenzano, L.; Galvelis, R.; Chen, P.-F.; Bennett, T. D.; Mellot-Draznieks, C.; Zicovich-Wilson, C. M.; Cheetham, A. K. Exceptionally Low Shear Modulus in a Prototypical Imidazole-Based Metal-Organic Framework. *Phys. Rev. Lett.* **2012,** *108*, 095502.
